# Haploinsufficiency for Core Exon Junction Complex Components Disrupts Embryonic Neurogenesis and Causes p53-Mediated Microcephaly

**DOI:** 10.1371/journal.pgen.1006282

**Published:** 2016-09-12

**Authors:** Hanqian Mao, John J. McMahon, Yi-Hsuan Tsai, Zefeng Wang, Debra L. Silver

**Affiliations:** 1 Department of Molecular Genetics and Microbiology, Duke University School of Medicine, Durham, North Carolina, United States of America; 2 Department of Pharmacology, University of North Carolina, Chapel Hill, North Carolina, United States of America; 3 Department of Cell Biology, Duke University School of Medicine, Durham, North Carolina, United States of America; 4 Department of Neurobiology, Duke University School of Medicine, Durham, North Carolina, United States of America; 5 Duke Institute for Brain Sciences, Duke University, Durham, North Carolina, United States of America; Stowers Institute for Medical Research, UNITED STATES

## Abstract

The exon junction complex (EJC) is an RNA binding complex comprised of the core components Magoh, Rbm8a, and Eif4a3. Human mutations in EJC components cause neurodevelopmental pathologies. Further, mice heterozygous for either *Magoh* or *Rbm8a* exhibit aberrant neurogenesis and microcephaly. Yet despite the requirement of these genes for neurodevelopment, the pathogenic mechanisms linking EJC dysfunction to microcephaly remain poorly understood. Here we employ mouse genetics, transcriptomic and proteomic analyses to demonstrate that haploinsufficiency for each of the 3 core EJC components causes microcephaly via converging regulation of p53 signaling. Using a new conditional allele, we first show that *Eif4a3* haploinsufficiency phenocopies aberrant neurogenesis and microcephaly of *Magoh* and *Rbm8a* mutant mice. Transcriptomic and proteomic analyses of embryonic brains at the onset of neurogenesis identifies common pathways altered in each of the 3 EJC mutants, including ribosome, proteasome, and p53 signaling components. We further demonstrate all 3 mutants exhibit defective splicing of RNA regulatory proteins, implying an EJC dependent RNA regulatory network that fine-tunes gene expression. Finally, we show that genetic ablation of one downstream pathway, p53, significantly rescues microcephaly of all 3 EJC mutants. This implicates p53 activation as a major node of neurodevelopmental pathogenesis following EJC impairment. Altogether our study reveals new mechanisms to help explain how EJC mutations influence neurogenesis and underlie neurodevelopmental disease.

## Author Summary

The mammalian neocortex is the brain structure responsible for higher cognition, abstract thought, and language. One process critical for brain development is neurogenesis, in which neural stem cell populations generate neurons. Alterations in neurogenesis can lead to neurodevelopmental disorders affecting brain size and function, such as microcephaly, in which the brain is significantly smaller than normal. Therefore, understanding the genes and processes controlling normal brain development is of strong clinical relevance. Here we studied proteins of the RNA binding exon junction complex, which are strongly implicated in several neurodevelopmental pathologies, but whose functions in brain development remain largely unknown. Using mouse models, we find that reduced levels of any of three essential proteins of this complex results in altered embryonic neurogenesis and microcephaly. We demonstrate that mutant mice show common alterations in p53 activation, expression of ribosomal components and splice variants for RNA processing factors. Interestingly we find that genetic suppression of p53 significantly rescues microcephaly in mutant mice. Given that patients harboring mutations in exon junction complex components present with neurodevelopmental deficits, our findings highlight molecular pathways which could underlie disease pathogenesis.

## Introduction

**Fig 1:**
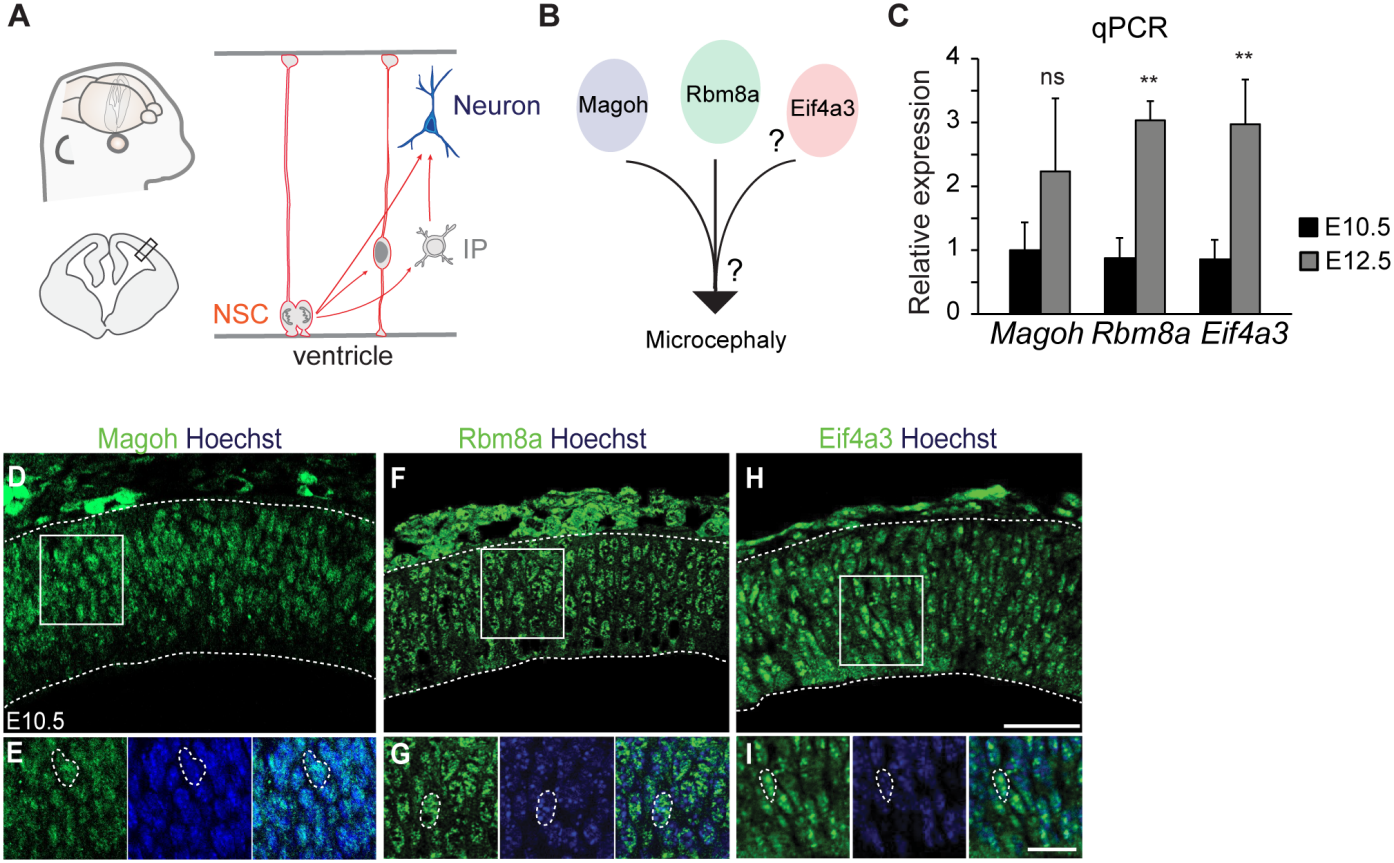
EJC components are co-expressed in neurogenesis. (A) Schematic of embryonic neurogenesis of the dorsal telencephalon. NSC, neural stem cell; IP, intermediate progenitor. (B) Two main questions posed in this study. 1. Does *Eif4a3* haploinsufficiency cause microcephaly? 2. Do EJC components regulate common pathways during neurogenesis? (C) qPCR of *Magoh*, *Eif4a3*, and *Rbm8a* mRNA levels in developing neocortices of indicated ages. qPCR was performed using a standard curve, with *Magoh* relative expression at E10.5 set to 1.0, and all expression levels normalized to *Gapdh*. (D-I) Immunofluorescence of E10.5 dorsal neocortices for Hoechst (blue), Magoh (D, E), Rbm8a (F, G), and Eif4a3 (H, I). (E, G, I) are high magnification images of D, F, H, respectively. Student’s *t* test, Error bars, S.D., **, *p*<0.01, ns = not significant. n = 3 biological replicates each age. Scale bars, D, F, H; 50 μm; E, G, I, 25 μm.

Proper function of the cerebral cortex, our brain structure responsible for higher cognitive functions, relies upon embryonic neurogenesis. During neurogenesis, neural stem cells (NSCs) generate excitatory neurons [1,2]. In mice the onset of neurogenesis is embryonic day (E) 10.5, when NSCs consist of neuroepithelial cells that primarily undergo self-renewal divisions. As development proceeds, neuroepithelial cells are replaced by radial glial cells that generate neurons either directly, or indirectly via new NSCs and intermediate progenitors (IPs) (Fig 1A) [3–5]. Defective neurogenesis impacts neuron production and can cause neurodevelopmental disorders such as microcephaly, in which brain size is severely reduced. To elucidate causes for such diseases requires a comprehensive understanding of how NSCs mediate proper brain development.

One level of control increasingly implicated in NSC function and disease is post-transcriptional regulation [6–8]. In particular, a set of RNA binding proteins associated with developmental pathologies of the cerebral cortex is the exon junction complex (EJC). The core EJC, composed of Rbm8a (Y14), Magoh, and Eif4a3 (Ddx48), influences mRNA splicing, translation, mRNA localization, and nonsense mediated decay (NMD), via direct interactions with both RNA and auxiliary proteins in the nucleus and cytoplasm [9–15]. Copy number variations of *RBM8A*, *EIF4A3*, and peripheral EJC components, are each strongly associated with neurodevelopmental phenotypes [16–18]. Moreover *RBM8A* and *EIF4A3* mutations cause TAR syndrome and Richieri-Costa-Pereira syndrome, respectively, both of which are associated with neurological deficits [19–22]. While altered EJC levels are significantly linked to neurodevelopmental diseases, the pathogenic mechanisms by which EJC impairment causes these disorders are largely unknown.

Recent studies from our lab have helped shed light on this question, with the discoveries that haploinsufficiency for either *Magoh* or *Rbm8a*, disrupts mouse cortical development. In these mouse models, both NSCs and IPs are depleted, neurons are ectopic, and there is massive apoptosis of neurons and progenitors, all leading to severe microcephaly [23–26]. We recently discovered that in *Magoh* mutants these neurogenesis phenotypes may be due in part to prolonged mitosis of NSCs [26]. Moreover, we identified Lis1 as one relevant Magoh downstream target during neurogenesis [23]. While these studies show *Magoh* and *Rbm8a* are essential for corticogenesis, it remains unknown if impairment of the third major EJC constituent, *Eif4a3*, causes microcephaly. Additionally, if all EJC components are required in the developing brain, it is unclear whether they function via common regulatory pathways. This information is critical to understand how EJC genes regulate brain development.

In this study we examined mice haploinsufficient for *Magoh*, *Rbm8a*, or *Eif4a3*, to expose mechanisms by which EJC dysfunction impacts cortical development. First, we generated a NSC-specific conditional *Eif4a3* mouse model to demonstrate that *Eif4a3* haploinsufficiency phenocopies the aberrant neurogenesis and microcephaly seen in *Rbm8a* and *Magoh* mutants. We then utilized transcriptomic and proteomic analyses to uncover common genetic pathways controlled by all 3 EJC components at the onset of neurogenesis. These include expression of factors associated with the ribosome, proteasome, and p53 signaling pathway. All 3 EJC mutants showed splicing alterations in RNA processing factors, implicating the EJC in regulating a network of RNA metabolism factors. Finally, we focus on one of these downstream pathways, p53, and show that *p53* ablation significantly rescues microcephaly of all 3 EJC mutants. Altogether our study reveals novel mechanisms to help explain how EJC deficiency disrupts neurogenesis, implicating elevated p53 signaling in the etiology of EJC-mediated neurodevelopmental pathologies.

## Results

### *Eif4a3* haploinsufficiency causes aberrant neurogenesis and microcephaly

We previously showed that NSC-specific haploinsufficiency for either *Magoh* or *Rbm8a* causes microcephaly in mice [23–25]. To understand whether common mechanisms contribute to microcephaly following depletion of EJC core components, we first sought to address the role of the third core EJC component, *Eif4a3*, in brain development (Fig 1B). We examined the expression profile of *Eif4a3* relative to *Magoh* and *Rbm8a* at early stages of cortical development. RT-qPCR showed that *Magoh*, *Eif4a3*, and *Rbm8a* are expressed in the developing neocortex and show parallel increases in expression as neurogenesis proceeds (Fig 1C). *In situ* hybridization revealed enriched *Eif4a3* expression in the proliferative ventricular and sub-ventricular zones of the E14.5 neocortex, where NSCs reside, in a similar pattern to *Rbm8a* and *Magoh* [23,24,27] (S1A Fig). Immunostaining showed that at the onset of neurogenesis (E10.5), EIF4A3 protein is expressed at detectable levels and is primarily localized within the nucleus, similar to MAGOH and RBM8A (Fig 1D–1I). Together, these analyses indicate that *Eif4a3*, *Magoh* and *Rbm8a* are co-expressed spatially and temporally in the developing mouse neocortex.

**Fig 2:**
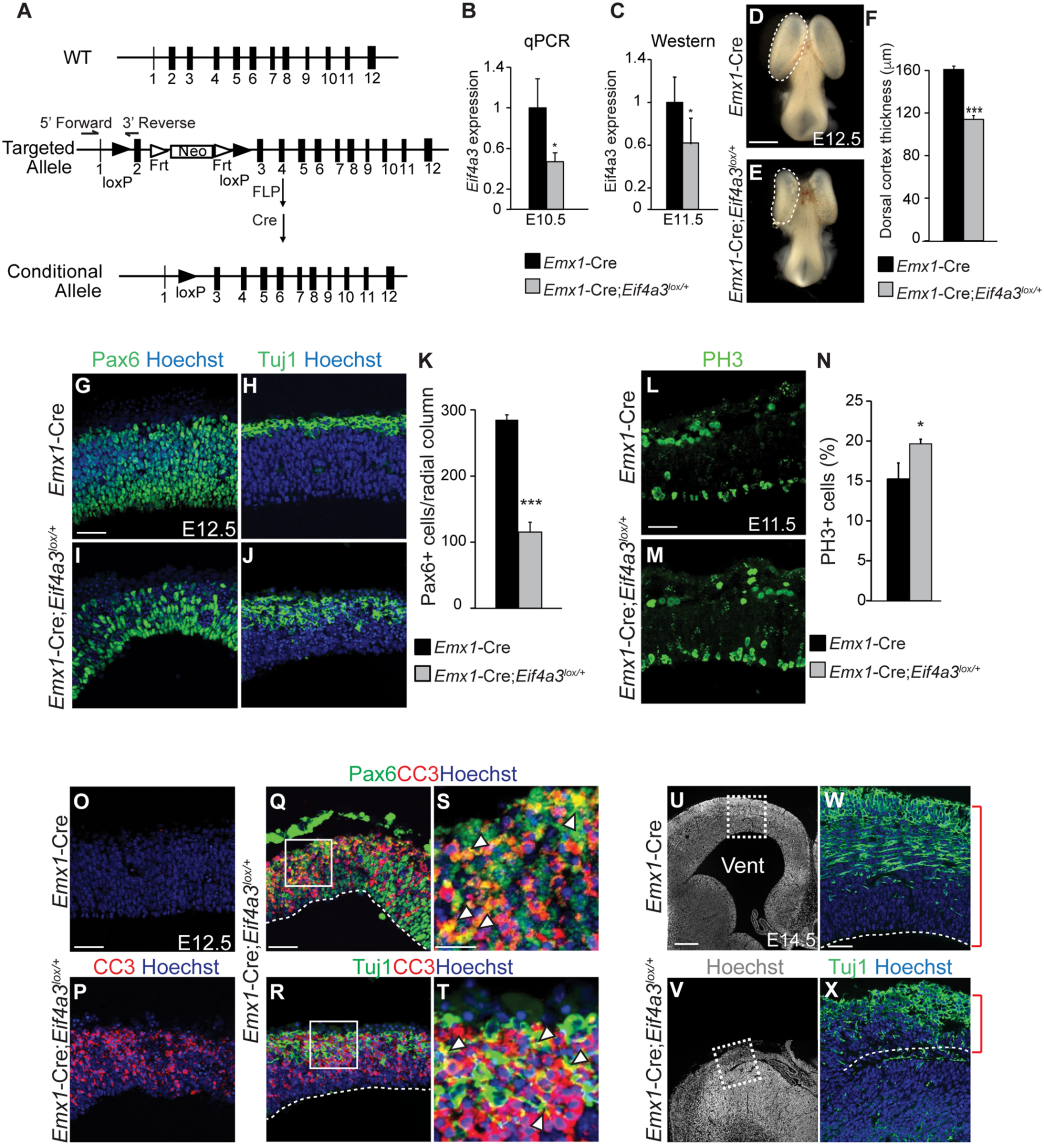
*Eif4a3* is required for embryonic neurogenesis and brain size. (A) Top, *Eif4a3* genomic mouse locus. Middle, targeted allele with 2 loxp sites (black arrowheads), *Neo* cassette, and 2 *FRT* sites (white arrowheads). Genotyping primers are indicated. Bottom, the conditional allele following FLP- and Cre-mediated recombination. (B) qPCR quantification of *Eif4a3* mRNA levels in E10.5 neocortices, following normalization using *Gapdh*. *Eif4a3* mRNA level of *Emx1*-Cre samples was set to 1.0. (C) Quantification of Eif4a3 protein levels in E11.5 dorsal cortices by densitometry of western blots, following normalization with α-Tubulin for loading. (D,E) Whole mount E12.5 *Emx1*-Cre and *Emx1*-Cre;*Eif4a3*^*lox*/+^ brains. Note the forebrain (dotted lines) is noticeably smaller in the *Eif4a3* mutant. (F) Quantification of cortical thickness of E12.5 *Emx1*-Cre and *Emx1*-Cre;*Eif4a3*^*lox*/+^ dorsal neocortices. (G-J) 4 different coronal sections from E12.5 *Emx1*-Cre (G,H) and *Emx1*-Cre;*Eif4a3*^*lox*/+^ (I,J) neocortices stained for Hoechst (blue), Pax6 (green, G,I) or Tuj1 (green, H,J). (K) Density of Pax6+ cells within 200 μm wide radial columns spanning the E12.5 cortices of indicated genotypes. (L, M) Images of E11.5 *Emx1*-Cre (L) or *Emx1*-Cre;*Eif4a3*^*lox*/+^ (M) cortices stained for PH3 (green). (N) Graph depicting percentage of all cells which are PH3-positive for indicated genotypes at E11.5. (O-T) E12.5 coronal sections from *Emx1*-Cre (O) and *Emx1*-Cre;*Eif4a3*^*lox*/+^ brains (P-T) stained for Hoechst (blue), CC3 (red), Pax6 (green, Q, S), and Tuj1 (green, R,T). S and T are high-magnification views of Q and R, respectively, as indicated. Arrowheads depict cells co-labeled for apoptotic and cell fate markers. (U-X) Coronal sections of E14.5 *Emx1*-Cre (U,W) and *Emx1*-Cre;*Eif4a3*^*lox*/+^ (V,X) cortices stained for Hoechst (white or blue) and Tuj1 (green). W and X are high-magnification images of U and V, respectively as indicated. Red brackets denote cortical thickness. Vent, ventricle. Student’s *t* test, *, *p*<0.05, ***, *p*<0.001. Error bars, S.D. n = 3 biological replicates each. Scale bars, D, E, 1 mm; G-J, L,M,O-R, W, X, 50 μm; S,T, 20 μm; U,V, 200 μm.

We generated a conditional mouse carrying a floxed allele of *Eif4a3* (*Eif4a3*^*lox/+*^) to assess the phenotype of *Eif4a3* deficiency in the developing brain (Fig 2A). *Eif4a3*^*lox/+*^ mice were crossed to *Emx1*-Cre, which drives Cre expression in NSCs of the dorsal neocortex beginning at E9.5 [28,29] (cre.jax.org). Genotyping of genomic DNA from *Emx1*-Cre;*Eif4a3*^*lox/+*^ mice confirmed the presence of predicted bands for both *wildtype* and *lox* alleles (S1B Fig). Following Cre recombination, exon 2 is excised to generate a transcript predicted to undergo NMD-mediated degradation. Consistent with this, *Eif4a3* mRNA and protein were reduced by about 50% in *Emx1*-Cre;*Eif4a3*^*lox/+*^ neocortices (Fig 2B and 2C). These data demonstrate *Eif4a3* can be efficiently depleted in the conditional haploinsufficient mouse model.

We next analyzed the impact of *Eif4a3* haploinsufficiency upon neurogenesis. At E12.5, *Emx1*-Cre;*Eif4a3*^*lox/+*^ cortices were markedly smaller at a whole mount level (Fig 2D and 2E) and 30% thinner when compared to control (*Emx1*-Cre) littermates (Fig 2F). PAX6-positive NSCs were significantly reduced in density in *Emx1*-Cre;*Eif4a3*^*lox/+*^ neocortices compared to control, an observation corroborated by western analysis (Fig 2G, 2I, 2K and S1C and S1D Fig). The depletion of NSCs was associated with a concomitant increased thickness of the TUJ1-positive neuronal layer (Fig 2H and 2J). These findings, smaller brain size, NSC depletion, and excessive neurons, demonstrate that *Eif4a3* haploinsufficiency phenocopies *Emx1*-Cre;*Rbm8a*^*lox/+*^ and *Emx1*-Cre;*Magoh*^*lox/+*^ neocortices [23,24]. We also previously showed that *Magoh*^*Mos2/+*^ germline mutant and *Emx1*-Cre;*Rbm8a*^*lox/+*^ NSCs exhibit significant mitotic defects [23,24,26]. Quantification of mitotic index using phospho-histone 3 (PH3) staining revealed increased mitotic index of E11.5 *Emx1*-Cre;*Eif4a3*^*lox/+*^ neocortices compared to control (Fig 2L–2N). Extensive apoptosis as evidenced by cleaved-caspase3 (CC3), is also associated with *Magoh* and *Rbm8a* haploinsufficient brains [23,24]. Similarly, we noted extensive apoptosis in *Emx1*-Cre;*Eif4a3*^*lox/+*^ neocortices (Fig 2O and 2P). Apoptosis was present in both neurons and NSCs throughout the dorsal cortex, similar to *Magoh* and *Rbm8a* mutants (Fig 2Q–2T). These early neurogenic phenotypes impacted brain structure. At E14.5, the dorsal telencephalon was largely absent in *Eif4a3* haploinsufficient brains (Fig 2U and 2V). Of the remaining dorsal telencephalon tissue found adjacent to the pallial-subpallial boundary, the cortex was extremely thinned and neurons were disorganized (Fig 2W and 2X). These phenotypes are highly similar to *Emx1*-Cre;*Rbm8a*^*lox/+*^ brains [24].

**Fig 3:**
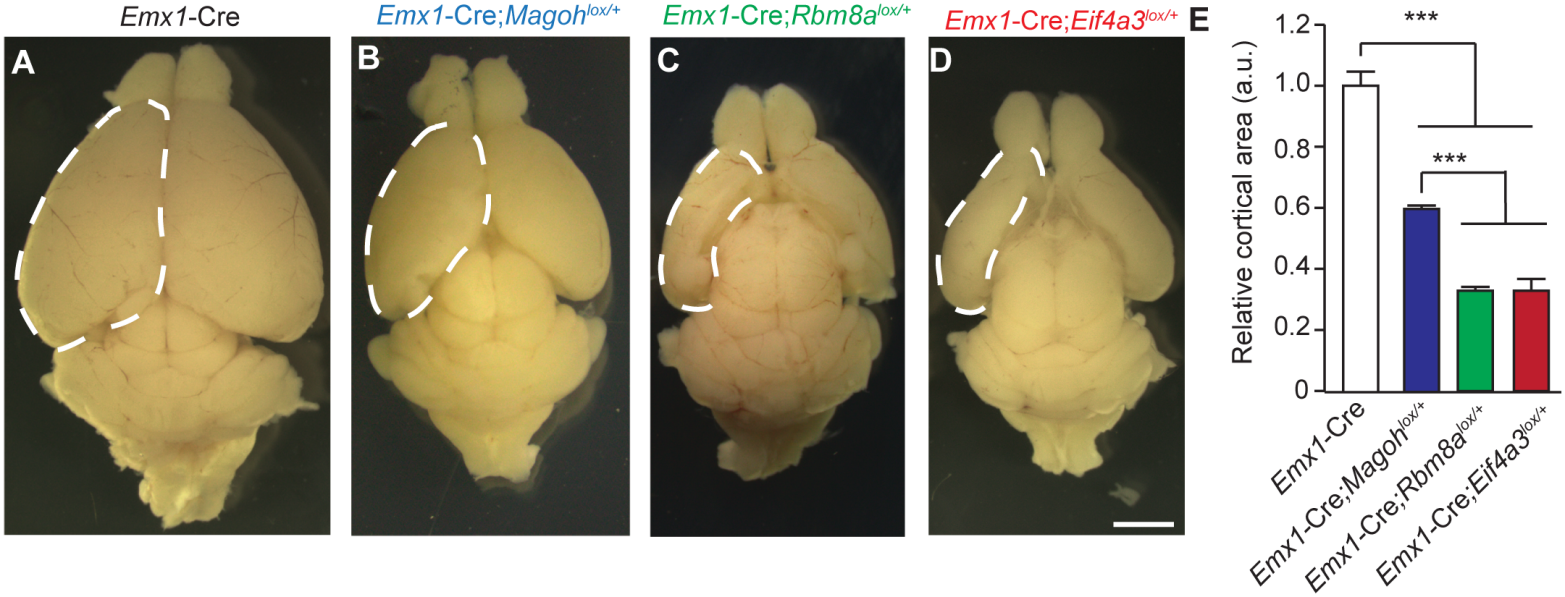
Haploinsufficiency of EJC components causes microcephaly. (A-D) Images of whole mount brains at P12 from indicated genotypes. Dotted lines denote dorsal cortex. (E) Quantification of relative dorsal cortical area in P12 brains of indicated genotypes. The area of *Emx1*-Cre brains was set to 1.0. ANOVA with Tukey posthoc, ***, *p*<0.001, Error bars, S.D. n = 3–4 biological replicates, Scale bar, A-D, 2 mm.

Surprisingly, despite the prevalent disruption of the developing neocortex, *Eif4a3*, *Rbm8a*, and *Magoh* conditional mutant mice survive into adulthood [24,25]. We evaluated postnatal (P) brain sizes of each EJC mutant. Comparison of P12 whole mount brains demonstrated significant reductions in all 3 EJC mutants (Fig 3A–3E). Both *Eif4a3* and *Rbm8a* haploinsufficiency caused severe microcephaly, with an average 70% reduction in cortical area of whole mount brains [24] (Fig 3E). The microcephaly phenotype of *Rbm8a* and *Eif4a3* mutant mice was significantly worse than *Magoh* haploinsufficient mice, which exhibited a 40% reduction [24,25]. This phenotypic difference may be due to redundant expression of a second *Magoh* homolog, whereas the other EJC components do not have identifiable homologs [30]. Together with our previous studies, these analyses indicate that Eif4a3, Magoh, and Rbm8a each control similar aspects of neurogenesis (NSC proliferation, number and apoptosis), and ultimately brain size.

### Transcriptome analysis of EJC mutants reveals alterations in expression levels of ribosomal, proteasome, and p53 signaling components

**Fig 4:**
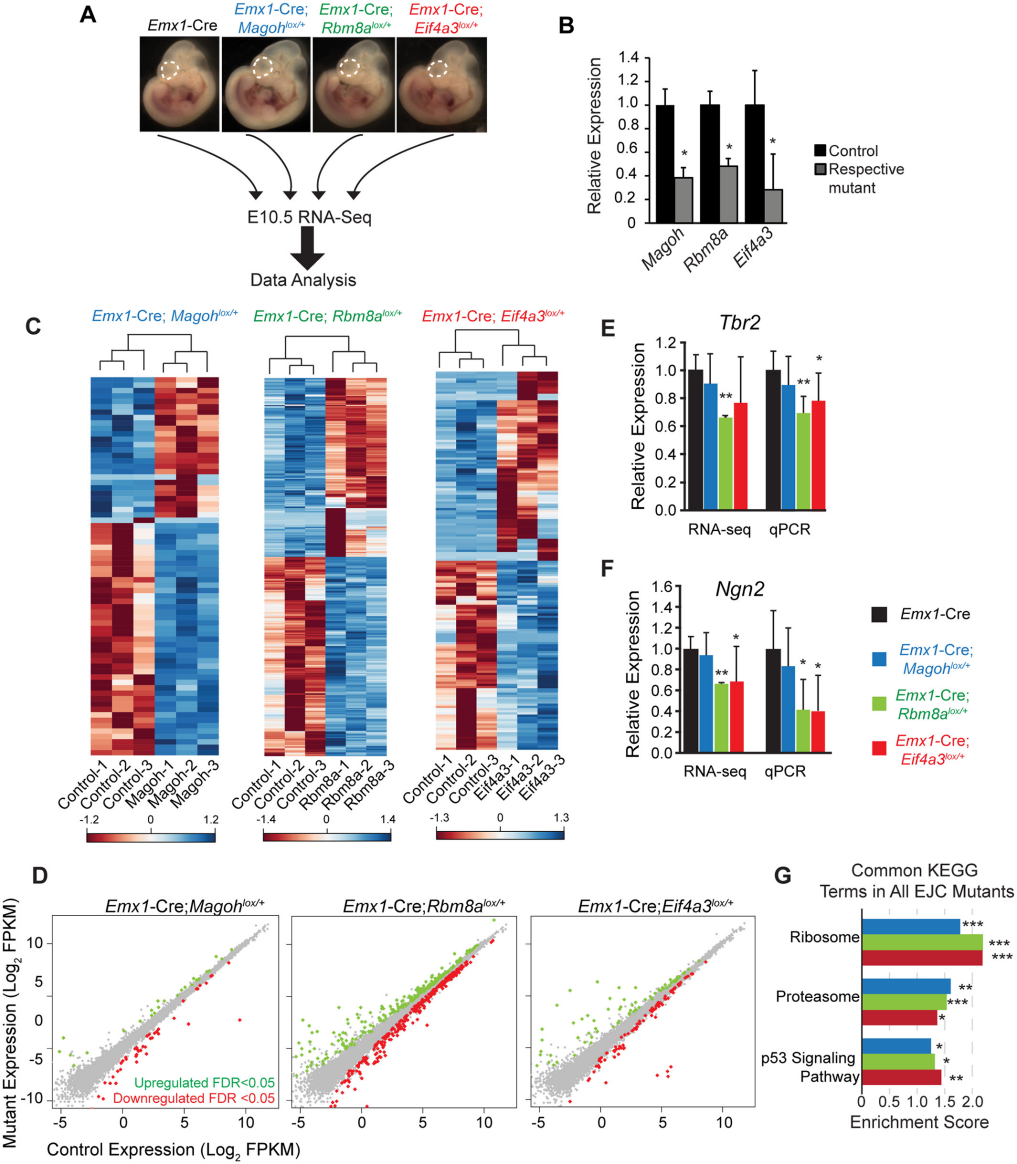
Transcriptome analyses of E10.5 *Magoh*, *Rbm8a*, and *Eif4a3* haploinsufficient cortices reveal common downstream pathways. (A) Diagrammatic overview of RNA sequencing analysis of E10.5 neocortices from indicated genotypes. (B) qPCR showing expression of *Magoh*, *Rbm8a*, and *Eif4a3* in their respective mutant E10.5 cortices. (C) Heatmaps showing z-score transformed normalized expression for all affected transcripts with an FDR corrected p-value, *q*< 0.05. Genes and samples were clustered using correlation distance with complete linkage. (D) Scatter plots of transcripts significantly upregulated (green dots) and downregulated (red dots) in E10.5 *Emx1*-Cre;*Magoh*^*lox/+*^, *Emx1*-Cre;*Rbm8a*^*lox/+*^, and *Emx1*-Cre;*Eif4a3*^*lox/+*^ cortices (q<0.05). (E, F) qRT-PCR validation at E11.5 compared to relative RNA-seq values of *Tbr2* (E) and *Ngn2* (F) in the indicated genotypes. For RNA-seq and qPCR, each control was normalized to 1.0 and compared to mutants. (G) Graph depicting common KEGG terms identified by GSEA analysis that were significant in all 3 EJC mutants, showing corresponding enrichment score. Student’s *t* test (B,E,F), Error bars, S.D. *, *p*<0.05, **, *p*<0.01, ***, *p*<0.001.

Given the overlapping expression patterns, common neurogenesis phenotypes, and vast literature connecting Magoh, Rbm8a, and Eif4a3, we hypothesized that these EJC components work together to influence cortical development. To test this, we aimed to identify molecular changes associated with early neurogenesis in each of the three EJC mutants. We performed transcriptome profiling of E10.5 neocortices from the following genotypes: *Emx1*-Cre, *Emx1*-Cre;*Rbm8a*^*lox/+*^, *Emx1*-Cre;*Magoh*^*lox/+*^, and *Emx1*-Cre;*Eif4a3*^*lox/+*^ (n = 3 biological replicates each) (Fig 4A). We focused on E10.5 for several reasons. This stage marks the beginning of neurogenesis when the neocortex is composed primarily of self-renewing neuroepithelial NSCs [4]. Moreover, it is just prior to the onset of severe defects in EJC mutants, and a stage when all 3 genes are reduced in their respective mutants, as evidenced by RT-qPCR of the RNA-sequencing samples (Fig 4B).

We examined global RNA changes in the 3 mutants relative to the control and to each other. Amongst the 18,465 detectable coding and non-coding transcripts expressed in the E10.5 control cortex, 2.9% were altered in *Emx1*-Cre;*Rbm8a*^*lox/+*^, 0.9% were altered in *Emx1*-Cre;*Eif4a3*^*lox/+*^, and 0.4% were altered in *Emx1*-Cre;*Magoh*^*lox/+*^ (FDR, q<0.05) (S1 Table). Hierarchical clustering of these significantly altered transcripts revealed segregation of control and mutant biological replicates for all 3 EJC mutants, as evidenced in heat maps (Fig 4C). Equivalent proportions of transcripts were upregulated and downregulated within individual EJC mutants (Fig 4D, S1 Table). We validated expression for several differentially expressed transcripts, *Tbr2*, *Ngn2*, *NeuroD6*, and *Gtse1*, using RT-qPCR, which showed similar trends to RNA-seq data (Fig 4E and 4F, S2B Fig). Despite the fact that the EJC binds a large fraction of expressed transcripts in immortalized cells [31–33], these experiments suggest EJC haploinsufficiency does not broadly impair transcript levels of E10.5 neocortices. This observation echoes previous microarray studies of germline *Magoh*^*Mos2/+*^ mutant brains [23], *Eif4a3* silenced *Xenopus* [34], and EJC *Drosophila* mutants [35].

We next assessed the extent to which transcripts overlapped amongst the EJC mutants, focusing only on the fraction of alterations which were highly significant (q<0.05). We noted extensive overlap in pairwise comparisons between individual mutants (S2A Fig). Of the 70 *Magoh* dependent transcripts, 87% were altered in *Rbm8a* mutants and 46% were altered in *Eif4a3* mutants. Of the 172 transcripts altered in *Eif4a3* mutants, 19% overlapped with *Magoh* mutants and 46% overlapped with *Rbm8a* mutants. Fisher’s exact tests demonstrated these overlapping changes were highly significant. In all 3 mutants, 31 transcript changes overlapped, which represents 6%, 18%, and 44% of all altered transcripts in the *Rbm8a*, *Eif4a3*, and *Magoh* mutants, respectively. As noted by Venn diagram, some transcript alterations were specific to individual mutants (S2A Fig). This was especially evident in *Rbm8a* and *Eif4a3* mutants, and suggests there could be roles for EJC components outside of the complex. Yet, taken together, these data support the notion that EJC components also work together to selectively affect mRNA levels at the onset of neurogenesis.

Given the significant overlap in transcript changes, we hypothesized *Magoh*, *Rbm8a*, and *Eif4a3* mutants influence common molecular and cellular pathways. To determine if this was true, we performed gene set enrichment assays (GSEA) Kyoto Encyclopedia and Genes and Genomes (KEGG) analysis on all 18,465 detectable genes from the transcriptome data, ranked by *p* value. Of those pathways significantly altered in all 3 EJC mutants, we discovered enrichment in only ribosome, proteasome, and p53 signaling (Fig 4G and S2 Table). This was also evidenced by enrichment plots and STRING analyses (S3A–S3I Fig). Closer inspection of the significantly altered transcripts within each KEGG category revealed extensive overlap amongst the 3 mutants (S3B, S3D and S3F Fig). Gene ontology (GO) analyses using GSEA further corroborated ribosomal alterations in all 3 haploinsufficient mutants (S2C Fig and S2 Table). The directionality and degree of expression changes in ribosome-encoding transcripts were consistent across all mutant mice (S2D Fig). Notably, inspection of only the significant transcript changes for each mutant (q<0.05) showed that ribosomal-associated transcripts made up 11.4%, 6.5% and 7.5% of *Magoh*, *Rbm8a*, and *Eif4a3* mutants, respectively. This indicates altered protein homeostasis pathways, including the ribosome, are shared early consequences of EJC haploinsufficiency.

**Fig 5:**
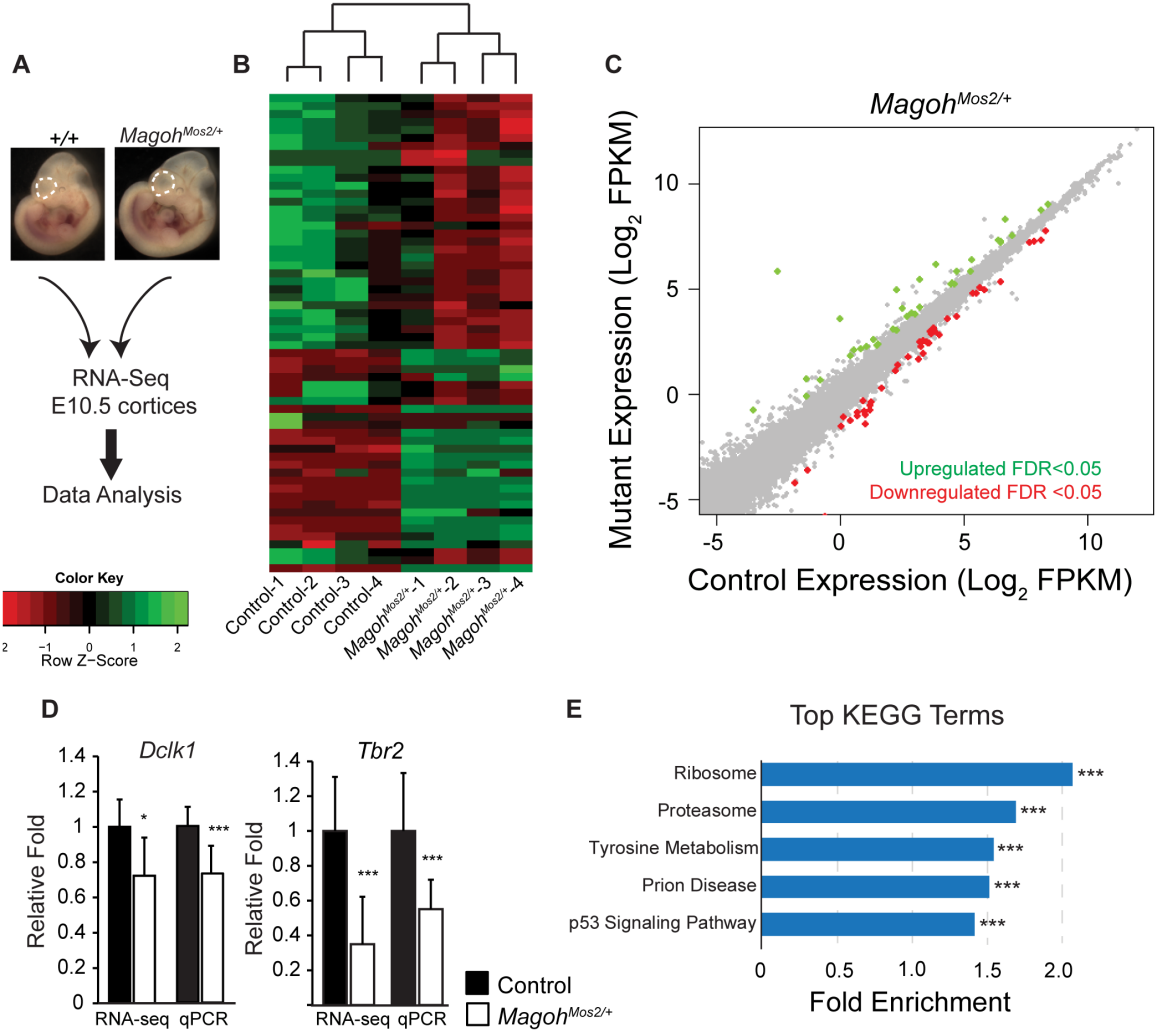
Transcriptome analyses of E10.5 *Magoh* germline haploinsufficient brains identifies alterations in ribosome and p53 signaling pathways. (A) Diagrammatic overview of RNA sequencing analysis of E10.5 neocortices (dotted lines) from indicated genotypes. (B) Heatmaps showing z-score transformed normalized expression for control and *Magoh*^*Mos2/+*^. Genes and samples were clustered using correlation distance with complete linkage. (C) Scatter plot of transcripts significantly upregulated (green dots) and downregulated (red dots) in E10.5 *Magoh*^*Mos2/+*^ cortices (*q*<0.05), n = 4 biological replicates each. (D) Validation and RNA-seq values for *Dclk1* and *Tbr2* in indicated E11.5 mutant dorsal neocortices. Controls were normalized to 1.0. (E) Graph depicting top ranked KEGG terms by GSEA analysis in *Magoh*^*Mos2/+*^ showing corresponding fold enrichment. Student’s *t* test (D), Error bars, S.D. *, *p*<0.05, ***, *p*<0.001.

To assess transcript regulation in an independent EJC model not reliant on Cre, we performed RNA-sequencing on E10.5 neocortices from control (C57BL/6J) and germline *Magoh* haploinsufficient mice (*Magoh*^*Mos2/+*^) (n = 4 biological replicates each) (Fig 5A). Hierarchical analysis revealed consistent expression changes in *Magoh*^*Mos2/+*^ compared to control littermates (Fig 5B). Amongst the 23,577 genes detected, only 80 (0.3%) transcripts were differentially expressed (q<0.05), and these were equivalently upregulated and downregulated (Fig 5C, S1 Table). RT-qPCR validation confirmed alterations in two transcripts, *Dclk1* and *Tbr2*, with similar trends to RNA-seq (Fig 5D). Changes were more dramatic than in *Emx1*-Cre;*Magoh*^*lox/+*^, consistent with a more severe impact of the *Magoh* germline deletion [23,25]. GSEA KEGG analysis of all detectable transcripts revealed significant enrichment for ribosome, proteasome, and p53 signaling components, amongst additional regulators of protein metabolism (Fig 5E and S4A Fig). GO analysis also detected ribosomes as a top altered category (S4C Fig). Of note, we observed overlap between *Emx1*-Cre;*Magoh*^*lox/+*^ and *Magoh*^*Mos2/+*^ transcripts within the ribosome, proteasome, and p53 categories (S4B Fig). Altogether, these transcriptome analyses from 4 independent mouse lines, including 2 models of *Magoh*, demonstrate that EJC haploinsufficiency influences a few common pathways including ribosome, proteosome and p53 signaling.

### Haploinsufficiency for *Magoh*, *Eif4a3*, and *Rbm8a* causes aberrant splicing of RNA regulatory proteins

**Fig 6:**
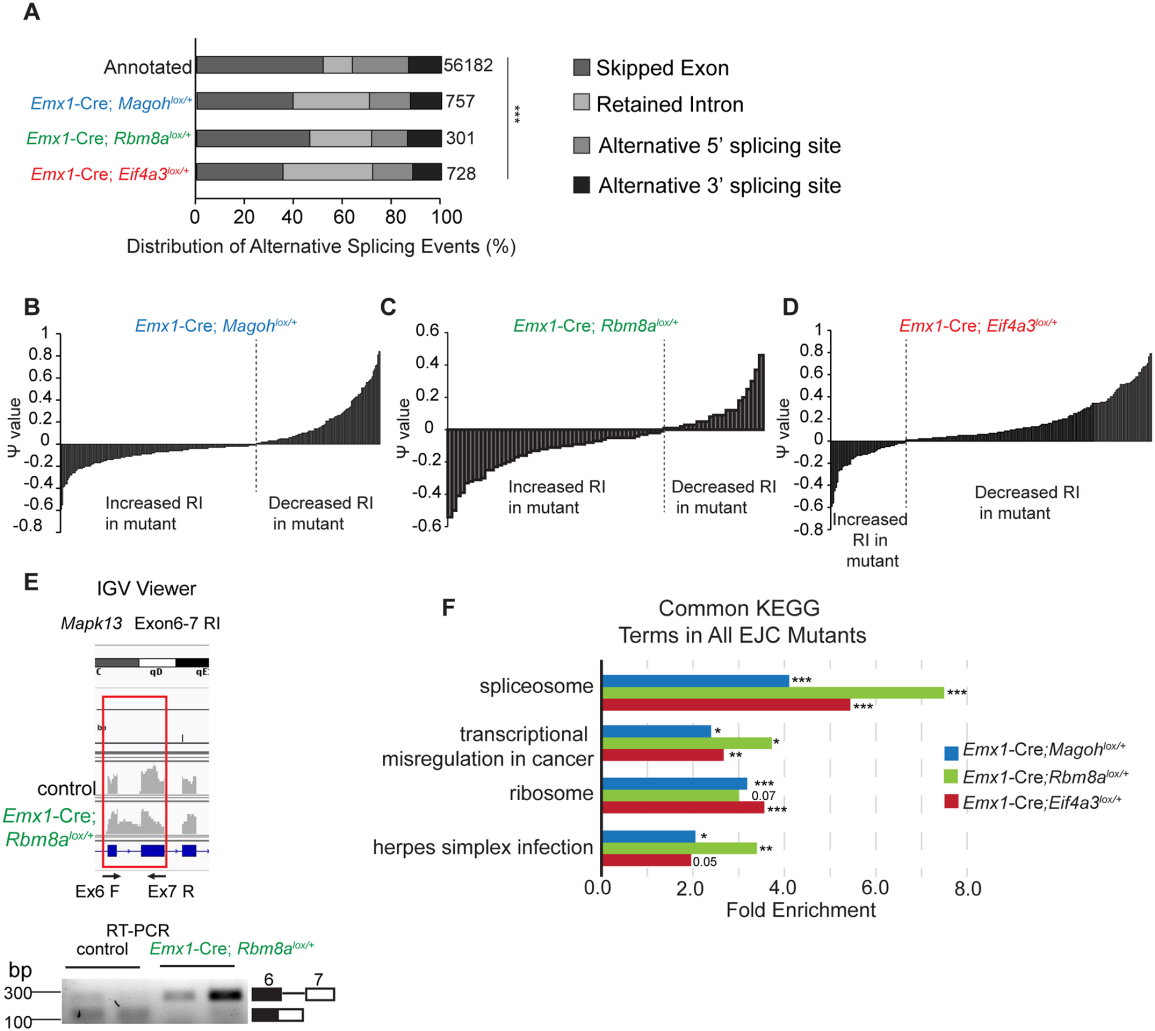
Haploinsufficiency for EJC components alters mRNA splicing of splicing regulators. (A) Bar graph showing alternative splicing events for each mutant relative to the control. (B-D) Clustered column graphs of the distribution of ψ values of all identified intron retention (RI) events in E10.5 dorsal cortices for each EJC mutant, using a threshold of 20 for Bayes factor. Ψ<0 indicates higher probability for the mutant to have intron retention when compared to the control. (E) Top: IGV view of increased *Mapk13* intron 6–7 reads in red frame. Primers indicated as arrows. Bottom: RT-PCR showing increased *Mapk13* RI isoforms in *Emx1*-Cre;*Rbm8a*^*lox/+*^ E11.5 dorsal cortices compared to the control. (F) Bar graph of common KEGG terms that were significant in all 3 EJC mutants, showing corresponding enrichment score. ANOVA (A), Modified fisher’s exact test (F), *, *p*<0.05, **, *p*<0.01, ***, *p*<0.001.

Given the requirement of the EJC in splicing and NMD, we next assessed splicing isoforms in the transcriptome data. Consistent with a published study in human cell lines [36], widespread splicing changes were evident in all 3 EJC mutants compared to control (S3 Table). We measured specific splicing events relative to all annotated alternative splicing (AS) events using Mixture-of-Isoforms (MISO) software [37,38]. Comparing the changed AS events to all annotated AS events, the distribution of AS types was significantly altered, with a 2–3 fold enrichment in retained intron (RI) events in all 3 EJC mutants (*p*<0.001) (Fig 6A). Amongst the RI events, 61%, 70%, and 23% were increased in *Magoh*, *Rbm8a*, and *Eif4a3* haploinsufficient mutants, respectively (Fig 6B, S3 Table). In *Emx1*-Cre;*Rbm8a*^*lox/+*^, 91% of RI events introduced a premature stop codon, which presumably leads to mRNA degradation through NMD (S3 Table). We validated several events, including *Mapk13* in E11.5 *Emx1*-Cre;*Rbm8a*^*lox/+*^ brains and *Fus* in *Magoh*^*Mos2/+*^ brains, noting alterations consistent with predictions (Fig 6E and S5A Fig). Thus, the enrichment of RI events could be due to inefficient NMD activity [12,14]. Consistent with previous findings that EJC *Drosophila* mutants cause increased RI events [39–41], our data suggest EJC components influence mRNA splicing in NSCs.

We next used bioinformatics analysis to determine if there are overlapping classes of splicing variants in the 3 EJC mutants. We performed KEGG analysis on those genes with significant alterations in splice variant expression (Bayes factor > 20) using the Database for Annotation, Visualization and Integrated Discovery (DAVID). These analyses showed common terms amongst all EJC mutants, including a significant enrichment of spliceosome (Fig 6F). GO analysis reinforced this finding, with enrichment of RNA regulatory categories including ribonucleoproteins and ribosomes (S4 Table). 51 identical alternative splicing events were predicted among all 3 EJC mutants. String analyses of these genes revealed two clusters for ribosome regulation and splicing regulation (S5B Fig). These data suggest that in addition to influencing transcript expression, EJC components have been co-opted to impact splicing of RNA regulators. Together, this implies an EJC dependent regulatory network that fine-tunes gene expression at the RNA level.

### Proteomic analyses reveal core EJC components influence protein levels of ribosomal components and RNA processing factors at the onset of neurogenesis

**Fig 7:**
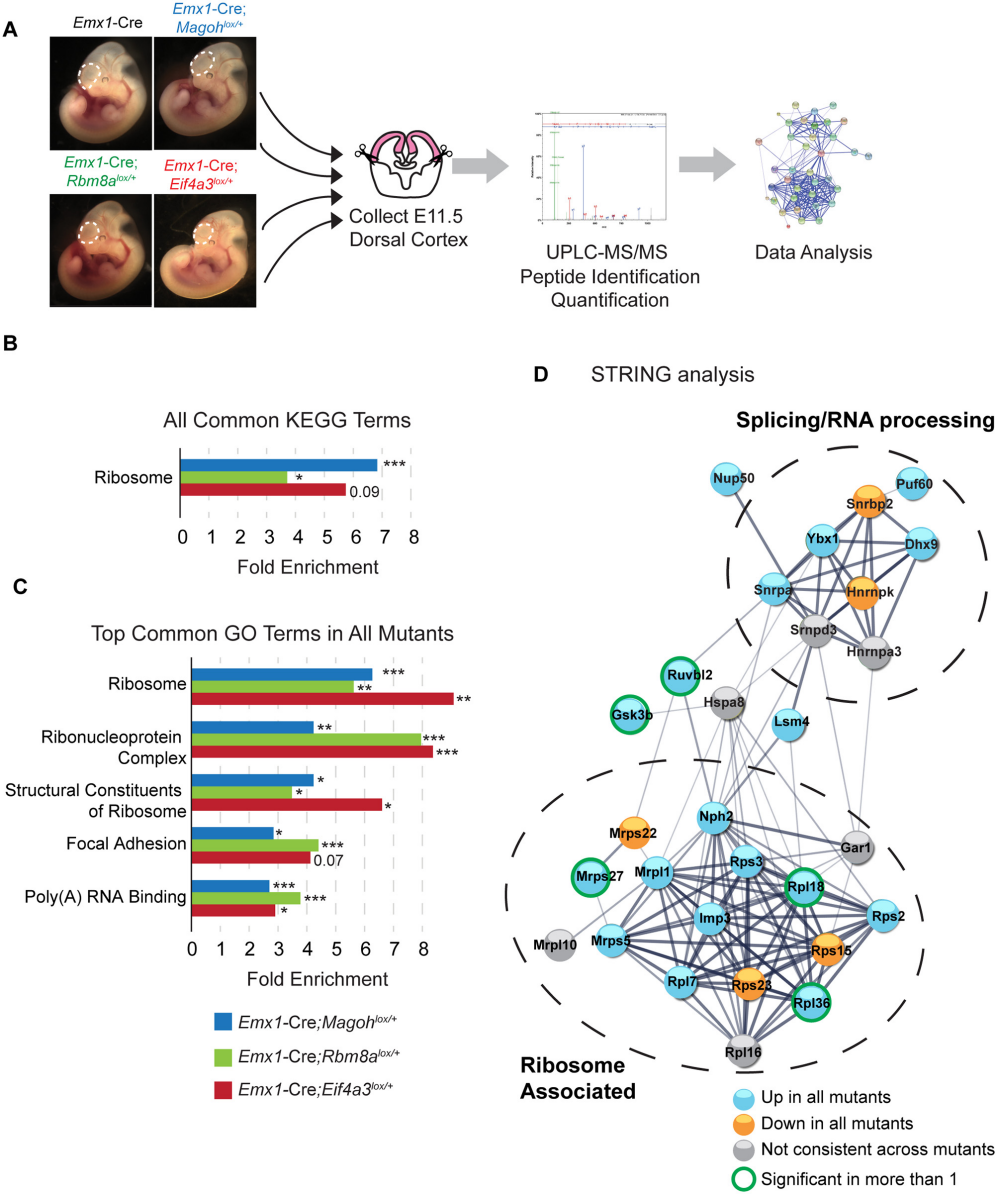
Proteomic analysis of E11.5 EJC mutant brains reveals alterations in levels of ribosome-associated proteins and ribonucleoproteins. (A) Diagrammatic representation of workflow to perform proteomic analysis of E11.5 dorsal cortices. (B, C) Bar graph of all common enriched KEGG terms (B) and top common GO terms (C) among all 3 EJC mutants showing corresponding fold enrichment and *P* values. (D) STRING network analysis of proteins within the broadest GO category, “Ribonucleoprotein Complex” altered in any of the 3 EJC mutants. Stronger associations are represented by thicker lines, and circles are colored based upon alteration in 1 or more mutants and level of significance. Two networks of splicing regulators and ribosome-associated proteins are detected. Modified fisher’s exact test, *, *p*<0.05, **, *p*<0.01, ***, *P*<0.001.

We next measured the proteomes of control, *Magoh*, *Rbm8a*, and *Eif4a3* haploinsufficient E11.5 neocortices using quantitative proteomic liquid chromatography/mass spectrometry (LC_MS/MS) analyses (n = 3 biological replicates each) (Fig 7A, S5 Table). We detected 3,587 proteins in the control and assessed relative levels of these proteins in each of the mutants. *Magoh*, *Eif4a3*, and *Rbm8a* haploinsufficiency led to significant alterations in 3.8%, 1.5%, and 4.3% of the detectable proteome, respectively (*p*<0.05). Consistent with our transcriptome analysis, the proteomes of the various mutants showed both overlapping and independent alterations (S6A Fig).

We next asked if there were common alterations amongst those proteins significantly altered in the 3 EJC mutants. Using KEGG DAVID analysis to examine only significant protein changes (*p*<0.05), we identified ribosomes as the only pathway enriched in all 3 EJC mutants, significant in 2 of the mutants (Fig 7B). GO analysis showed components of ribosomes and ribonucleoprotein complexes amongst the most significantly enriched categories (Fig 7C, S6 Table). We performed STRING analysis of all altered proteins in the EJC mutants within the largest GO term, “Ribonucleoprotein Complex,” which included ribosome components and splicing factors (Fig 7D). This analysis reinforced strong regulatory networks present amongst proteins downstream of the EJC, and the consistent directional changes evident in all 3 mutants. Closer inspection of all significant protein changes within each mutant showed that ribosomal proteins made up 7.9%, 5.8%, and 7.5% of *Magoh*, *Rbm8a*, and *Eif4a3* mutant changes, respectively. A large fraction of ribosomal proteins changed consistently across all 3 mutants, showing up and down regulation at the protein level (S6B Fig). Altogether these genomic and proteomic analyses support the notion that ribosome and ribonucleoprotein alterations are major early defects associated with EJC deficiency in the developing brain.

### Activation of p53 is a major contributor to microcephaly of EJC mutant mice

**Fig 8:**
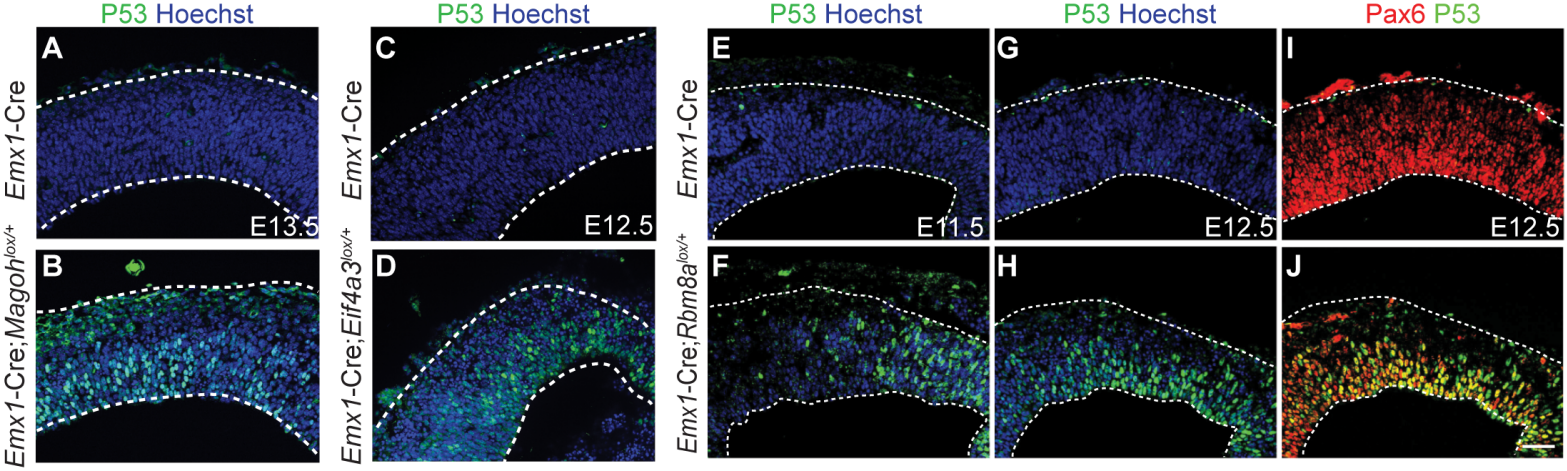
EJC haploinsufficiency induces P53 activation. (A-J) Coronal sections of cortices from E13.5 *Emx1*-Cre (A), E13.5 *Emx1*-Cre;*Magoh*^*lox/+*^ (B), E12.5 *Emx1*-Cre (C, G, I), E12.5 *Emx1*-Cre;*Eif4a3*^*lox/+*^ (D), E11.5 *Emx1*-Cre (E), E11.5 *Emx1*-Cre;*Rbm8a*^*lox/+*^ (F), and E12.5 *Emx1*-Cre;*Rbm8a*^*lox/+*^ (H,J) embryonic cortices stained for Hoechst (blue), P53 (green), and Pax6 (red), with co-localization indicated in yellow. Sections were demarcated with dotted lines. Each image is representative of at least 3 independent biological samples. Scale bar, A-J, 50 μM.

The omics analyses pointed to several common pathways that are dysregulated at the onset of neurogenesis, and suggested candidate molecules that could be relevant for EJC mutant phenotypes. We hypothesized that p53 signaling, in particular, was a major contributor to EJC-mediated microcephaly. Activated p53 is a key regulator of apoptosis and defective cell cycle progression [42], two major phenotypes of EJC mutant brains. Moreover, p53 target transcripts were upregulated in all 3 conditional EJC mutants and *Magoh* germline mutant (Figs 4 and 5, S1 Table). Additionally, a correlation has been previously observed in p53 transcript changes in *Magoh* germline brains and induced radiation [43]. Altogether these data suggest p53 activation may be a common critical node in disease pathogenesis following EJC impairment. We thus probed the relationship between EJC haploinsufficiency and p53 signaling, by assessing p53 nuclear accumulation in embryonic brain sections, as a proxy for pathway activation [26]. Haploinsufficiency for *Magoh*, *Rbm8a*, and *Eif4a3* led to a significant accumulation of p53 in the VZ compared to control brains, which showed no evidence of p53 accumulation (Fig 8A–8J). Western blotting confirmed accumulation of p53 protein in *Eif4a3* mutant cortices (S7A Fig). P53 activation was evident in E11.5 *Rbm8a* mutants (Fig 8E and 8F), prior to the onset of apoptosis [24], and was specifically enriched in PAX6-positive NSCs (Fig 8I and 8J). This demonstrates that p53 is activated in EJC haploinsufficient NSCs.

**Fig 9:**
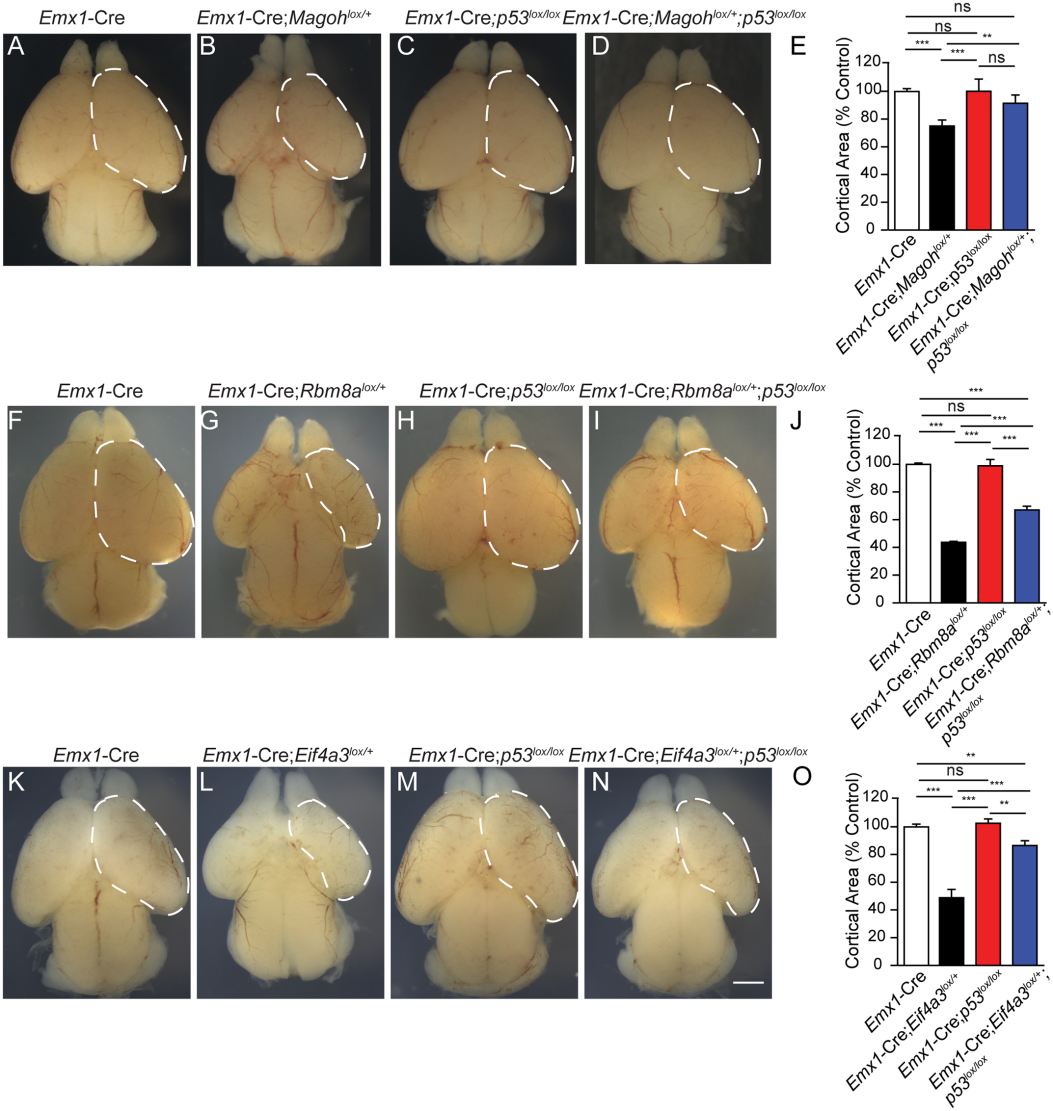
Loss of *p53* partially rescues microcephaly of *Magoh*, *Rbm8a*, and *Eif4a3* haploinsufficient mutants. (A-D, F-I, and K-N) Whole mount brains of E18.5 embryos with indicated genotypes. (E, J, O) Quantification of cortical area in E18.5 embryonic brains with indicated genotypes. Dotted lines demarcate the dorsal cortical areas measured. The surface area of littermate control brains was set to 100. ANOVA with Tukey posthoc, **, *p*<0.01, ***, *p*<0.001, NS, not significant. Error bars, S.D. n = 3–9 biological replicates each. Scale bars, A-D, E-I, and K-N, 1 mm.

We hypothesized that p53 activation contributes to microcephaly phenotypes of all 3 EJC mutants. To directly assess this, we crossed *Emx1*-Cre;*Magoh*^*lox/+*^, *Emx1*-Cre;*Rbm8a*^*lox/+*^, and *Emx1*-Cre;*Eif4a3*^*lox/+*^, onto a *p53*^*lox/lox*^ null background. We collected E18.5 embryos and measured cortical area. Compared to control, *Emx1*-Cre;*p53*^*lox/lox*^ did not alter brain size (Fig 9A, 9C, 9F, 9H, 9K and 9M). As expected, cortical area was significantly reduced in mice haploinsufficient for *Magoh*, *Rbm8a*, or *Eif4a3*, to a similar degree seen in adults (Compare Fig 9B, 9G and 9L to Fig 3). Strikingly, for all 3 EJC mutants the microcephaly was significantly, albeit partially, rescued in a *p53* mutant background (Fig 9D, 9I and 9N). Amongst the 3 EJC mutants, the extent of p53-mediated rescue varied and was most effective in the least severe microcephaly mutant, *Magoh* (Fig 9E, 9J and 9O). These data indicate that p53 activation is a major cause of microcephaly in all 3 EJC mutants. Our data also suggest that for *Rbm8a* and *Eif4a3*, additional p53 independent factors likely contribute to the reduced brain size.

To elucidate the nature of the p53-mediated rescue we examined apoptosis and neuron number. Amongst the 3 core EJC components, reduced *RBM8A* levels are the most strongly associated with human microcephaly [19,21,44]. Given this clinical relevance, we focused our analysis on the *Rbm8a* mutant. As p53 is essential for induction of apoptosis, we first assayed whether *p53* ablation rescued apoptosis in the *Rbm8a* mutant. As predicted, CC3 immunostaining revealed complete rescue of apoptosis in E12.5 *Emx1-*Cre*;Rbm8a*^*lox/+*^;*p53*^*lox/lox*^ brains (S7B–S7D Fig). Thus p53 activation promotes apoptosis downstream of *Rbm8a*.

**Fig 10:**
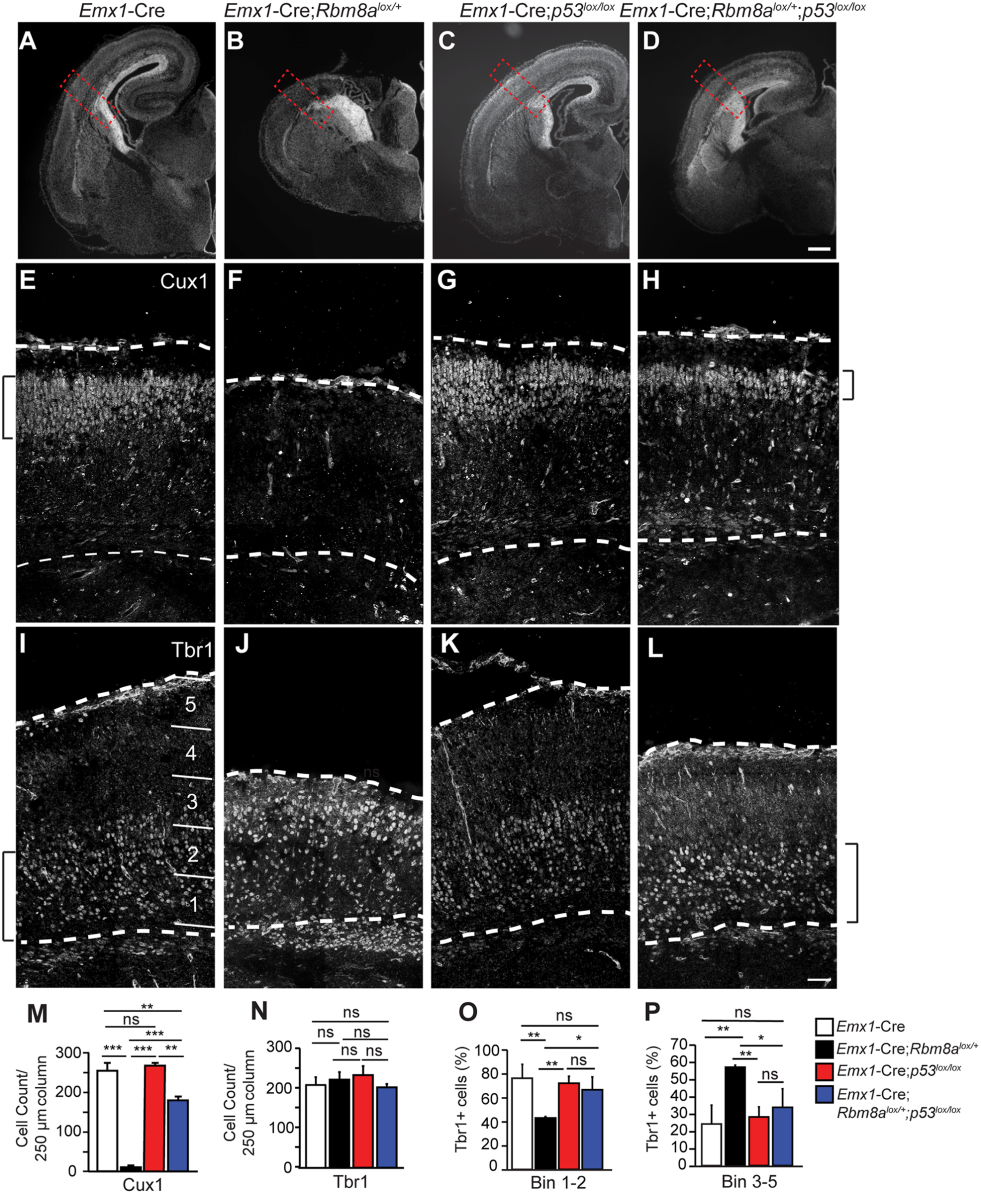
Loss of *p53* partially rescues neuron number and distribution associated with *Rbm8a* haploinsufficiency. (A-D) Coronal sections of E18.5 brains of indicated genotypes stained with Hoechst (white). (E-L) Regions of coronal sections indicated in (A-D, red dotted square) stained for Cux1 (E-H) and Tbr1 (I-L). (M, N) Quantification of Cux1+ (M) and Tbr1+ (N) density within a 250 μm radial column for indicated genotypes. (O, P) Bar graphs depicting density of Tbr1+ neurons in VZ/SVZ (bin 1–2, O) and cortical plate (bin 3–5, P) of indicated genotypes. Bins were quantified as indicated in I. Brackets denote general boundaries of Cux1 and Tbr1 layers. ANOVA with Tukey posthoc *, *p*<0.05, **, *p*<0.01, ***, *p*<0.001, ns, not significant. Error bars, S.D. n = 2–3 biological replicates each. Scale bars, A-L, 50 μm.

We next examined neuronal layers of E18.5 brains (Fig 10A–10D). As we have previously shown [24], *Rbm8a* mutant brains are missing most of their pallium (Fig 10B). In the *Emx1-*Cre*;Rbm8a*^*lox/+*^;*p53*^*lox/lox*^ brains, the pallium is restored, consistent with the rescue of apoptosis (Fig 10D). We asked if *p53* loss impacts neuronal layers, focusing on the tissue adjacent to the pallial-subpallial boundary which is still present in *Rbm8a* mutants [24]. We quantified both deep and superficial neuronal markers which are generated at early and late stages of neurogenesis, respectively [4]. As predicted, Cux1+ neurons (layer II/III) were nearly ablated in *Emx1-*Cre*;Rbm8a*^*lox/+*^ brains, compared to control or *p53* alone (Fig 10E–10G). In contrast, in *p53*;*Rbm8a* compound mutant brains Cux1+ neuron number was largely rescued (Fig 10H and 10M). Another marker of both superficial and some deep layer neurons, Satb2, was reduced in *Emx1-*Cre*;Rbm8a*^*lox/+*^, but partially rescued in a *p53* null background (S7E–S7I Fig)[45,46]. We also examined earlier born deep layer Tbr1+ neurons (Fig 10I–10L). As we previously described [24], in *Emx1-*Cre*;Rbm8a*^*lox/+*^ brains Tbr1 number is normal but distribution is skewed basally (Fig 10I, 10J, 10K and 10N–10P). This is consistent with our previous finding that at early stages of development, Tbr1 density is increased in *Rbm8a* mutants, perhaps due to increased neuron production [24]. In *Emx1-*Cre*;Rbm8a*^*lox/+*^*;p53*^*lox/lox*^ brains, aberrant Tbr1+ neuron distribution was restored to normal (Fig 10L, 10O and 10P). These analyses show that in *Rbm8a* mutants, p53 activation influences the number and distribution of neurons generated at different stages of neurogenesis, and plays a particularly important role in genesis of upper layer neurons. Taken together, our data implicate p53 activation as a key node in the microcephaly pathology following EJC impairment.

## Discussion

The EJC is a central regulator of mRNA metabolism, yet how its molecular roles translate into physiological functions relevant for disease has been poorly understood. Here we used mouse haploinsufficiency models for the 3 core EJC components to demonstrate their common requirements for neurogenesis and proper brain size. We employed transcriptomics and proteomics to identify converging molecular pathways regulated by the EJC at the onset of neurogenesis. Our unbiased analyses demonstrate that reduced levels of *Magoh*, *Eif4a3*, or *Rbm8a* lead to altered expression of ribosomal components, splicing changes, and aberrant p53 signaling. We focused on the p53 pathway, demonstrating that aberrant p53 activation is a major contributor to EJC-mediated microcephaly. Given that human mutations in EJC components are associated with neurodevelopmental diseases, our study suggests these pathologies may be due in part to aberrant p53 activation.

### EJC controls gene expression in neural stem cells

Our study elucidates several layers of EJC-dependent gene expression in the developing neocortex. Whereas EJC-dependent targets are known in *Drosophila* and immortalized cells [35,36], our study is the first to discover EJC-dependent gene expression in a mammalian stem cell population. We demonstrate that EJC haploinsufficiency alters only a small fraction of the transcriptome, and these changes are disproportionately enriched for ribosomal proteins, proteasome components, and p53 signaling. Thus, the EJC may be especially important in regulation of protein homeostasis machinery. We also find the 3 core EJC proteins converge in regulating alternative splicing events. In particular we identify aberrant intron retention events which are suggestive of roles in mRNA splicing and NMD, and are consistent with genomic studies of EJC depletion in *Drosophila* and mammalian cells [36,39–41]. Notably these splicing changes are enriched for both spliceosomal and ribosomal components. Alterations in ribosomes are also observed at the protein level. Altogether, these analyses indicate the EJC is integral to an RNA regulation network controlling neurogenesis.

These findings raise several fascinating questions. Although we focused on common EJC regulatory pathways, our data also highlight there are unique targets of individual EJC components. In future studies it will be of interest to consider potential independent roles for EJC components outside of the complex in neurogenesis. Another interesting question is how the EJC differentially regulates its targets in individual cells. For example, although we measured genomic changes in tissue that is mainly composed of 1 cell type, neuroepithelial progenitors, observed transcript and splicing differences could be attributed to progenitors in different cell cycle states. Moreover, it will be of interest to determine if the same pathways are regulated by the EJC in non-*Emx1*-derived cell types.

### P53 attenuation rescues microcephaly caused by EJC haploinsufficiency

We demonstrate that EJC mutant mice all exhibit profound microcephaly, which is significantly rescued by *p53* deletion. Detailed analysis of *Rbm8a* mutants reveal that *p53* attenuation partially restores superficial neuron number and distribution of deep layer neurons. Thus, the dramatic loss of upper layers in *Rbm8a* mutants is due, in part, to p53 activation. At least 2 scenarios could explain this rescue. P53 induction of apoptosis may severely reduce both neuron and progenitor number, particularly at later stages when upper layers are produced. Aberrant p53 activation may also influence stem cell divisions and thus their progeny. Our lab previously showed increased mitotic index in *Magoh* and *Rbm8a* mutant NSCs [23,24,26]. Mitotically delayed *Magoh* mutant NSCs preferentially produce neurons and apoptotic progeny, at the expense of NSCs [26]. We find that p53 activation is evident at E10.5, which precedes the onset of mitotic defects at E11.5 and E12.5. Given this sequence of events, it is tempting to speculate that aberrant p53 activation may influence progenitor (and ultimately neuron) number by delaying mitosis. Future experiments will be useful for evaluating if this relationship is correlative or causal.

How might p53 be activated by EJC dysfunction? It is plausible that ribosomal alterations contribute to p53 activation, as evidenced in many examples from the literature for genes controlling ribosome biogenesis [42,47–51]. Alternatively, p53 could be activated independent of the ribosome, as seen in the pancreas [52]. The EJC could also directly regulate RNA metabolism of p53 pathway components, as has been observed in splicing of apoptotic regulators [53]. The mechanisms contributing to p53 activation in EJC models are a topic of future interest.

### Beyond p53: other alterations associated with EJC haploinsufficiency

For *Eif4a3* and *Rbm8a* mutants, p53 rescue was incomplete, suggesting there must be additional EJC-dependent pathways mediating early stages of neurogenesis. Our analyses implicate several promising candidates. Reduced expression of canonical neurogenesis regulators, including *Ngn2*, *Tbr2*, and *NeuroD6*, could contribute to cell fate changes in the neocortex. All 4 EJC mutants also showed alterations in components of the proteasome, indicating that the EJC could influence neurogenesis by regulating protein homeostasis. Our data also identify ribosomal alterations at the transcriptome, splicing, and proteomic level, suggesting ribosome regulation could contribute to EJC-dependent microcephaly. Indeed, human genetic studies suggest that ribosome biogenesis defects cause neurodevelopmental diseases [54]. Of the significant (FDR<0.05) changes in 3 different mutants, ribosomal transcripts made up 5–11%, well above the fraction expressed in progenitors, a finding which is reinforced with unbiased GSEA analysis.

How might the EJC influence expression of ribosomal components? The EJC could directly regulate ribosome biogenesis. Indeed, ribosome biogenesis defects are seen in *Fal1p S*. *cerevisiae* mutants [55,56] and *Eif4a3* siRNA-depleted mammalian cells [56–58]. Alternatively, highly expressed genes, which include both ribosomal and proteasome components, could be especially sensitive to EJC levels. Another possible explanation for our results is that ribosome alterations are an indirect result of overall cellular stress. This idea is supported by the observation that some ribosome-encoding transcripts are not altered in all 3 mutants. It is also notable that ribosomal transcripts at E10.5 were nearly universally upregulated, whereas one day later the proteins were differentially altered. This could be due to differences in RNA versus protein regulation or could suggest compensatory responses to restore ribosomal levels in the brain. Understanding the nature of how the EJC influences ribosome stoichiometry, and how this may influence microcephaly, will be an important question for the future.

### Roles for the EJC in neurogenesis and neurodevelopmental disorders

Mutations and copy number variations in core and peripheral EJC components are strongly associated with neurodevelopmental deficits in humans, yet the etiology of these pathologies is poorly understood. Microdeletions and duplications of a 15-gene locus containing *RBM8A* are associated with microcephaly, macrocephaly, autism, and epilepsy [19,20]. Compound inheritance of this deletion and a regulatory *RBM8A* mutation is responsible for TAR syndrome, a congenital malformation of blood and skeletal systems which can also present with neurological deficits [21]. Moreover, regulatory *EIF4A3* mutations cause a craniofacial disorder presenting with learning and language disabilities [22]. Intriguingly, both craniofacial and neurodevelopmental anomalies are associated with disruption of p53 signaling and ribosomal impairments [49,50,59,60]. It is notable the EJC downstream splicing changes include several genes, such as *RPL10*, which are mutated in patients with neurodevelopmental disorders [60]. Thus, it is interesting to consider whether some of the expression changes we have identified in mouse models may contribute to EJC disease etiology.

Altogether, based on our discoveries, we propose aberrant p53 signaling contributes to the pathology of EJC related disorders and that modifications of p53 signaling may be of potential therapeutic interest. It is tempting to speculate that EJC diseases could be considered as ribosomopathies. Going forward, the EJC haploinsufficient mouse mutants we have generated provide valuable models for understanding the etiology of microcephaly and dissecting cell autonomous requirements in NSCs. Future studies using ubiquitous knockout of EJC components may help to further model other disease manifestations. In summary, our findings demonstrate new mechanisms to explain how EJC haploinsufficiency causes microcephaly, which has implications for understanding physiological functions of the EJC in the developing brain and in disease pathogenesis.

## Materials and Methods

### Ethics statement

All experiments were performed in agreement with the guidelines from the Division of Laboratory Animal Resources from Duke University School of Medicine and IACUC.

### Mouse husbandry and generation of conditional *Eif4a3* allele

Plug dates were defined as embryonic day (E) 0.5 on the morning the plug was identified. The conditional targeting vector for ES cell targeting was designed and generated by the Transgenic Facility at Duke University Cancer center. Positive ES clones were selected by performing long-range PCR of both arms. For long-range PCR of 5’ arms, the following conditions were used: 94°C X 1 min (1X); 98°C X 10 s, 60°C X 15 s, 68°C X 8.5 min (40X); 72°C X 10 min. 5’ F1:GTCCCAGAAATATCAGTGAGAATC; 5’ R1:CTTGTCATCGTCGTCCTTGTAGTC. For long-range PCR of 3’ arms, the following conditions were used: 94°C X 2 min (1X); 98°C X 10 s, 60°C X 15 s, 68°C X 2.5 min (40X); 72°C X 10 min. Positive clones were electroporated into CD1 blastocysts, and the resulting chimeras were mated to C57BL/6J females to obtain germ-line transmission. For genotyping *Eif4a3*^*lox*^ mice, the following conditions were used: 94°C X 3 min (1X); 94°C X 15 s, 62°C X 20 s, 72°C X 30s (30X); 72°C X 10 min (1X). 5’ forward: CTTGCAGTTGTCTTTCTGCGG; 3’ Reverse: CACACATGGCGATCCGCTCG. The following strain was acquired from Jackson labs: *Emx1-Cre* (B6.129S2-*Emx1*^*tm1*^(cre)Krj/J).

### Western blot and RT-qPCR analyses

E10.5 neocortices and E11.5 dorsal cortices were collected from *Emx1-*Cre, *Emx1-*Cre*;Eif4a3*^*lox*/+,^ *Emx1-*Cre*;Rbm8a*^*lox*/+^, and *Emx1-*Cre*;Magoh*^*lox*/+^ mice and lysed in RIPA lysis buffer with protease inhibitors (Pierce, Rockford, IL). Cortical lysates were run on 4–20% pre-casted SDS–Polyacrylamide gels (Bio-Rad). For Pax6 and P53 blots, stain free gels were used for total protein normalization. Gels were transferred onto nitrocellulose membranes and blotted using the following primary antibodies: rabbit anti-Eif4a3 (1:200, Santa Cruz), rabbit anti-Pax6 (1:1,000, Millipore), rabbit anti-p53 (1:1,000, Leica) and mouse anti-α-Tubulin (1:10,000, Sigma). Blots were developed using ECL reagent (Pierce). Densitometry was performed using ImageJ. Final values were quantified by normalizing EJC protein levels to loading controls (1:10,000, Tubulin, Sigma) or UV-induced Stain-free pre-casted gel (Bio-Rad), and analyzed for significance using a Student’s *t* test. For qPCRs, whole neocortices from E10.5 and dorsal neocortices of E11.5, and E12.5 and E14.5 embryos were collected from C57BL/6J (wild-type), *Emx1-*Cre, *Emx1-*Cre*;Eif4a3*^*lox*/+,^ *Emx1-*Cre*;Rbm8a*^*lox*/+^, and *Emx1-*Cre*;Magoh*^*lox*/+^ embryos and RNA was extracted using Trizol reagent (Invitrogen) followed by the RNeasy kit (Qiagen). cDNA was prepared according to the iScript kit (Bio-Rad). qPCR was performed in triplicates using Taqman probes (Life Technologies): *Rbm8a* (Mm04214345_s1), *Eif4a3* (Mm00836350_g1), *Magoh* (Mm00487546_m1), *Ngn2* (Mm00437603_g1), *Tbr2* (Mm01351984_m1), *Dclk1* (Mm00444950_m1) and *Gapdh* (4352339E). Sybr Green iTaq (Biorad) was performed with primers designed for *Gste1* (5’Forward-CCAGAGCAAAGAGGACCAAG and 3’ Reverse-CCGTGAGAACTTTGGGGTTA), *NeuroD6* 5’ Forward-GCCTCAATGATGCTCTGGACAA and 3’ Reverse- CTCTTGCCAATCCTCAGAATTTCAG), and *β-Actin* (5’ Forward- CCTTCTTGGGTATGGAATCCTG and 3’ Reverse- GTTGGCATAGAGGTCTTTACGG). For wild-type samples at different developmental stages, semi-quantitative qRT-PCR was performed. A standard curve was generated with a 5 serial 10-fold dilution of cDNA from an independent E14.5 wildtype embryo. Final values were normalized to *Gapdh* loading control. For E10.5 control and conditional mutant samples, comparative qRT-PCR was performed. Values were normalized to *Gapdh* control. For each genotype, 3 embryos were examined, a student’s *t* test was run to determine the significance. For all experiments, 3 biological samples for each genotype were used.

### Immunohistochemistry and quantification of tissue sections

Brains were fixed overnight in 4% paraformaldehyde (PFA) at 4°C, followed by submersion in 30% sucrose until sinking, as previously described [23]. Brain cryostat sections (20 μm) were prepared and stored at -80°C until use. Sections were permeabilized with 0.25% TritonX-100 for 10 min and blocked with MOM block reagent (Vector laboratories) for 1 hour at room temperature (RT). Sections were incubated with primary antibodies for 2 hours at RT or overnight at 4°C. Sections were then incubated in species appropriate secondary antibodies and Hoechst for 15 min at room temperature. The following primary antibodies were used: rabbit anti-Magoh (1:200, Proteintech), rabbit anti-Rbm8a (1:200, Bethyl), rabbit anti-Eif4a3 (1:200, Bethyl), rabbit anti-CC3 (1:200; Cell Signaling), rabbit anti-Pax6 (1:1,000; Millipore); rabbit anti-PH3 (1:200, Millipore), rabbit anti-Satb2 (1:1000; Abcam), anti-Cux1 (1:250, Santa Cruz); mouse anti-Pax6 (1:50; DSHB); rabbit anti-p53 (1:250, Leica), mouse anti-TuJ1 (1:400; Covance). The following secondary antibodies were used: Alex Fluor 488, Alex Fluor 568, Alex Fluor 594 (1:200–400; Invitrogen) and Hoechst (1:1000; Invitrogen). High magnification images were captured using a Zeiss Axio Observer Z.1 microscope coupled with an apotome. Cortical thickness was measured with Zen software. Quantifications were performed using ImageJ. A minimum of 3 sections from anatomically comparable regions per embryo and 3 biological replicates from control and mutants were measured/quantified.

### RNA-Seq, splicing and bioinformatics

Control and EJC mutant embryonic neocortices were dissected at E10.5. Samples were flash-frozen in liquid nitrogen and stored at -80°C until further treatment. RNA was extracted with Trizol (Invitrogen) followed by micro-RNeasy kit (Qiagen) according to manufacturer’s protocol. The library was generated with Kapa stranded mRNA-seq Kit. The fragmented poly-A RNAs were sequenced using Illumina Hi-Seq 2000 double end sequencing with 100nt length. RNA-seq data was processed using the TrimGalore toolkit (http://www.bioinformatics.babraham.ac.uk/projects/trim_galore) which employs Cutadapt to trim low quality bases and Illumina sequencing adapters from the 3’ end of the reads [61]. Only pairs where both reads were 20 nt or longer were kept for further analysis. Reads were mapped to the NCBIM38r73 version of the mouse genome and transcriptome using the STAR RNA-seq alignment tool [62]. Reads were kept for subsequent analysis if they mapped to a single genomic location. Gene counts were compiled using the HTSeq tool (http://www-huber.embl.de/users/anders/HTSeq/). Only genes that had at least 10 reads in any given library were used in subsequent analysis. Normalization and differential expression was carried out using the EdgeR Bioconductor package with the R statistical programming environment [63]. The exact test method was used to identify differentially expressed genes between the different mouse genotypes. Inspection of reads using integrative genomics viewer (IGV) software confirmed altered regulation of pseudogenes. Heatmaps were prepared for z-score transformed normalized expression for genes with an FDR, q<5%. To calculate significant overlap for Venn diagrams the following criteria were used: genes must with a q<0.05 and using a Fisher’s Exact Test for overlap between any two conditions. For alternative splicing analysis, Mixture-of-isoforms (MISO)[38] model was used to analyze RNA-Seq data and estimate the percent of splicing isoforms (Ψ values, for ‘Percent Spliced Isoform’), and the differentially spliced events are identified using a stringent filter (bayes-factor >20). The program was run with pooled samples of 3 biological replicates to reduce sampling biases. Validation of RI events was performed by RT-PCR with cDNA prepared from E11.5 dorsal cortices of control and EJC mutant embryos. The following primers were used: *Fus* Ex6 Forward: GGCCAAGATCAGTCCTCTATGAGT, *Fus* Ex8 Reverse: CATGACGAGATCCTTGATCCCGA, *Mapk13* Ex6 Forward: GCAACCTGGCTGTGAATGAA, and *Mapk13* Ex7 reverse: CTGGTTGTAATGCATCCAGCTG.

### Pathway analyses for RNA seq and proteomics

For bioinformatic analysis of RNAseq Gene Set Enrichment Analysis (GSEA) was performed by creating a pre-ranked list of all detected transcripts, ranked by 1 minus the p value. The ranked list was imported into the GSEA software (GSEA v2.2.2, Broad Institute) and analyzed using the pre-ranked gene list function. The data bases used were KEGGv5.1 and GOv5.1. Common GSEA terms were cross compared among the 3 mutant strains and plotted according to their normalized enrichment score. The statistical test utilized by GSEA is the Kolmogorov-Smirnov statistical test. Venn diagrams include all genes that were identified as enrichment by the GSEA analysis within a given KEGG term. For splicing and proteomics analysis, we determined enriched pathways by assessing only significant hits (splicing analysis: MISO, Bayes factor >20 and proteomics, p<0.05). DAVID Annotation, Visualization and Integrated Discovery v6.7 was used to analyze significant changes by KEGG and gene ontology (GO) analysis (including biological process, molecular function, and cellular component). Significance of enrichment in GO term analyses was calculated using the p value function given from a modified Fisher’s exact test by the DAVID database. For splicing analysis, STRING (Search Tool for the Retrieval of Interacting Genes/Proteins) analysis was carried out with transcripts show significant splicing changes (Bayes>20) in all 3 EJC mutants. For proteomic analysis, STRING was carried out with significantly changed (*p*<0.05) proteins in the “ribonucleoprotein complex” GO term. All components not connected to other genes/proteins were not included in figures.

### Proteomics and bioinformatics

We performed relative quantitation proteomic study using the Duke Proteomics Core Facility. E11.5 dorsal cortices were dissected in cold PBS and flash-frozen in liquid nitrogen. Samples were stored in -80°C until use. 200 μl of 8M urea in 50 mM ammonium bicarbonate was added to E11.5 dorsal cortices. Samples were subjected to 3 rounds of probe sonication for 5s each with an energy setting of 30%. Samples were then centrifuged at 12,000g and 4°C for 5 minutes. All samples were run by LC/MS/MS and total ion current was used to normalize sample loading for final analysis. Samples were supplemented with 800 μl of 50 mM ammonium bicarbonate to reduce the urea concentration to 1.6M. Samples were reduced with 10 mM DTT at 80°C for 15 min and then alkylated at 25 mM iodoacetamide at room temperature for 30 min. Trypsin (1.7 μg) was added to each sample and allowed to proceed for 18 hr at 37°C. Samples were then acidified with 6 μl of TFA and subjected to a C18 cleanup using the 50 mg (1 cc) C18 Sep-Pak columns (Waters). After elution, the samples were spun to ~50% dryness in the vacuum centrifuge, frozen, and lyophilized to dryness. Samples were randomized in their run order and QC samples were run periodically throughout the acquisition window. Samples were initially resuspended in 12 μl of 1% TFA/2% acetonitrile with 10 fmol/μl yeast alcohol dehydrogenase. To create a “QC pool” sample to assess analytical reproducibility, 3 μl of each sample was removed and pooled. Quantitative LC/MS/MS was performed on 2 μl of each sample, using a nanoAcquity UPLC system (Waters Corp) coupled to a Thermo QExactive Plus high resolution accurate mass tandem mass spectrometer (Thermo) via a nanoelectrospray ionization source. Briefly, the sample was first trapped on a Symmetry C18 300 mm × 180 mm trapping column (5 μl/min at 99.9/0.1v/v water/acetonitrile), after which the analytical separation was performed using a 1.7 μm Acquity BEH130 C18 75 mm × 250 mm column (Waters Corp.) using 90-min linear gradient of 5 to 40% acetonitrile with 0.1% formic acid at a flow rate of 400 nl/min with a column temperature of 55°C. Data collection on the QExactive Plus mass spectrometer was performed in a data-dependent acquisition (DDA) mode of acquisition with a r = 70,000 (@ m/z 200) full MS scan from m/z 375–1600 with a target AGC value of 1e6 ions followed by 10 MS/MS scans at r-17,500(@ m/z 200) at a target AGC value of 5e4 ions. Following the 12 LC-MS/MS analyses, data were imported into Rosetta Elucidator v3.3 (Rosetta Biosoftware, Inc), and all LC-MS/MS runs were aligned based on the accurate mass and retention time of detected ions (“features”) which contained MS/MS spectra using PeakTeller algorithm (Elucidator). A mean normalization of the high confidence identified peptide features excluding the highest and lowest 10% of the identified signals (i.e. a robust mean normalization) was then employed. The relative peptide abundance was calculated based on area-under-the-curve (AUC) of aligned features across all runs. Database searching was performed within Mascot Server v2.5 (Matrix Science) and annotated using the Peptide Teller algorithm within Rosetta Elucidator at a peptide false discovery rate of 1%. Proteins representing membrane, nuclear and cytoplasmic fractions were present in the data.

## Supporting Information

S1 Fig Analysis of conditional *Eif4a3* haploinsufficient mutant. (A) *In situ* hybridization of *Eif4a3* in sagittal E14.5 mouse section, showing enrichment in the ventricular and sub-ventricular zones (arrowheads) relative to the cortical plate (CP). Images are from www.genepaint.org (Visel et al. 2004). (B) Representative PCR genotyping result from *Emx1*-Cre (control) and *Emx1*-Cre;*Eif4a3*^*lox*/+^ mice. Note a single band (432 bp) in control and two bands (432 bp and 490 bp) in *Emx1*-Cre;*Eif4a3*^*lox*/+^. (C) Representative western blot of Pax6. (D) Quantification of Pax6 expression from E10.5 cortical lysates. Error bars, S.D, n = 3 biological replicates each.
(TIF)

S2 Fig RNA seq Validation and analysis of EJC mutants. (A) Venn Diagrams showing the overlap of significant transcript changes (*q*<0.05) among the 3 EJC mutants. For pairwise comparisons between datasets, the percent of overlapping transcript changes within each mutant is shown, along with associated *p* values. (B) qPCR validation of *NeuroD6* and *Gtse1* mRNA expression in indicated E11.5 mutant cortices. For RNA-seq and qPCR, each control was normalized to 1.0 and compared to mutants. (C) Bar graph of top common enriched GO terms identified with GSEA analysis among all 3 EJC mutants, showing corresponding fold enrichment and P values. (D) Plot of all ribosomal protein transcripts for EJC mutant RNA seq. Student’s t-test (B). Error bars, S.D, *, *p*<0.05, **, *p*<0.01, ***, *p*<0.001.
(TIF)

S3 Fig GSEA analysis of transcriptome data of EJC mutants. (A, C, E) Enrichment plots from GSEA KEGG analysis for Ribosome (A), Proteasome (C) and p53 signaling (E) terms. (B, D, F) Venn diagrams of overlapping enriched genes between EJC mutants for the Ribosome (B), Proteasome (D), and p53 signaling (F) terms. (G, H, I) STRING analysis of the genes enriched in the Ribosome (G), Proteasome (H), and p53 signaling (I) pathways.
(TIF)

S4 Fig GSEA analysis of *Magoh*^*Mos2/+*^ transcriptome data. (A) Enrichment plots from GSEA KEGG analysis for Ribosome, Proteasome, and p53 signaling terms. (B) Venn diagrams of overlapping enriched genes between *Emx1-Cre;Magoh*^*lox/+*^ and *Magoh*^*Mos2/+*^ mutants for the Ribosome, Proteasome, and p53 signaling terms. (C) Top enriched GO terms from GSEA analysis of *Magoh*^*Mos2/+*^ transcriptome. ***, *p*<0.001.
(TIF)

S5 Fig Splicing Analysis of *Magoh*, *Rbm8a*, and *Eif4a3* haploinsufficient cortices. (A) Gel image showing detection of increased *Fus* Ex6-8 RI events in E11.5 *Magoh*^*Mos2/+*^ mutants compared to 2 litter mate controls. (B) STRING analysis including genes predicted to show identical, significant splicing changes in all 3 EJC mutants. Common genes that are not connected with any other genes by STRING analysis were not included. Stronger associations are represented by thicker lines. Note two networks of splicing regulation (cyan) and Ribosome/Translation (orange).
(TIF)

S6 Fig Proteomic Analysis of EJC mutants. (A) Venn diagrams of overlapping enriched proteins altered between EJC mutants *(p*<0.05). *(*B) Bar graph depicting all ribosomal protein changes relative to control showing similar trends in all 3 EJC mutants (control levels are set to 0).
(TIF)

S7 Fig P53 rescue of EJC neurogenesis phenotypes. (A) Representative western blot for p53 in *Emx1-*Cre and *Emx1-*Cre;*Rbm8a*^*lox/+*^ E11.5 dorsal cortex. (B-D) Immunostaining for CC3 in control (B), *Emx1*-Cre;*Rbm8a*^*lox/+*^*;p53*^*lox/+*^ (C) and *Emx1*-Cre;*Rbm8a*^*lox/+*^*;p53*^*lox/lox*^ (D). (E-H) Representative images of Satb2 immunostaining in E18.5 cortex from *Emx1*-Cre, *Emx1-*Cre;*Rbm8a*^*lox*/+^, *Emx1*-Cre;*p53*^*lox/lox*^ and *Emx1*-Cre;*Rbm8a*^*lox/+*^*;p53*^*lox/lox*^. (I) Quantification of Satb2+ cells from E-H. ANOVA with Tukey posthoc. Error bars, S.D. *,*p*<0.05, **,*p*<0.01, ***,*p*<0.001, Scale bar, 50 μm.
(TIF)

S1 Table Transcriptome analysis of EJC mutants. (XLSX)

S2 Table Transcriptome analysis GO terms. (XLSX)

S3 Table Splicing analysis of EJC mutants. (XLSX)

S4 Table Splicing analysis GO terms. (XLSX)

S5 Table Proteomic analysis of EJC mutants. (XLSX)

S6 Table Proteomic GO terms. (XLSX)
