# Supplementary figures and images for: Haploinsufficiency for Core Exon Junction Complex Components Disrupts Embryonic Neurogenesis and Causes p53-Mediated Microcephaly
Source: PLoS Genet. 2016 Sep 12;12(9):e1006282. doi: 10.1371/journal.pgen.1006282 (PMC5019403; doi:10.1371/journal.pgen.1006282)

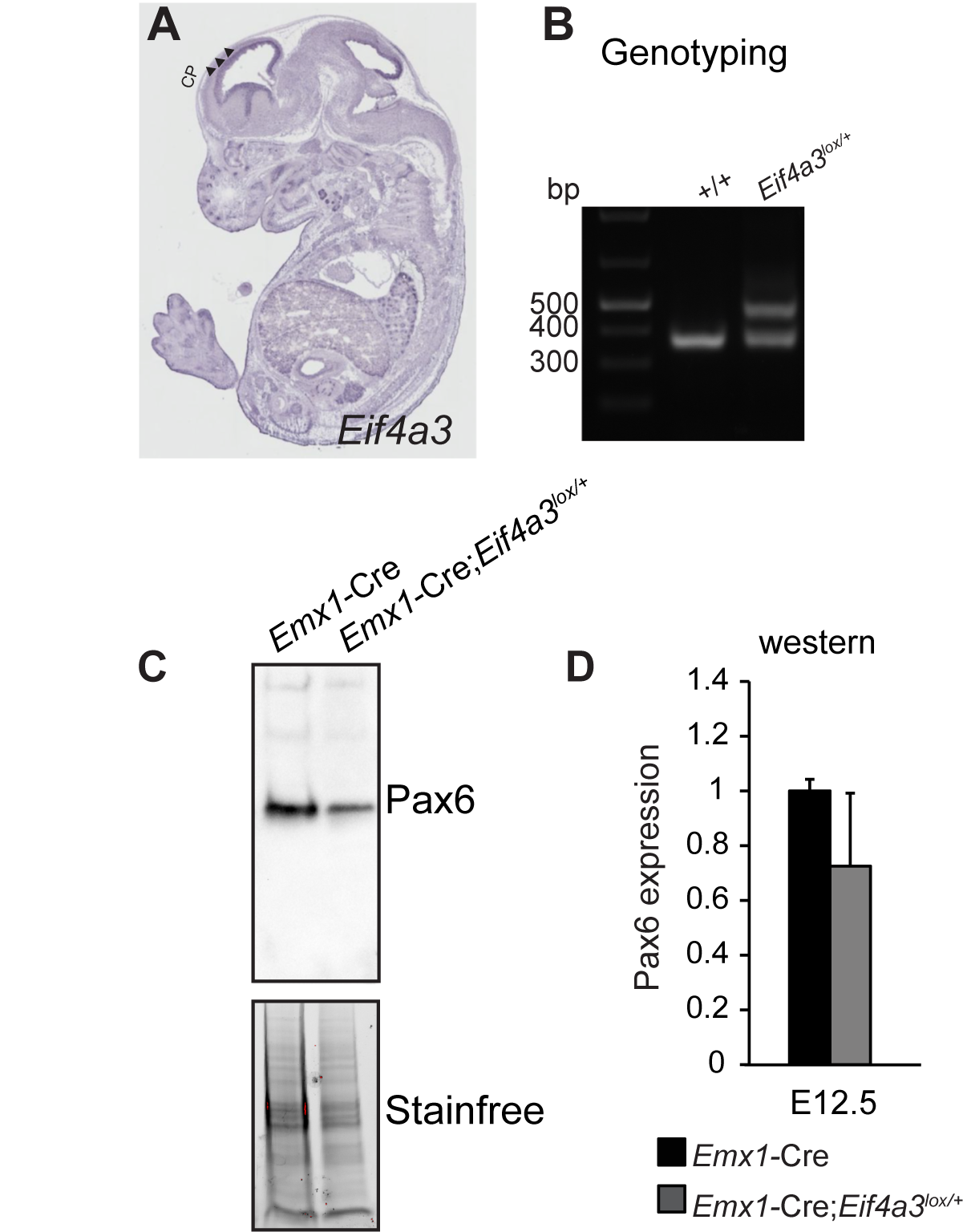

Supplement: S1 Fig — (A) In situ hybridization of Eif4a3 in sagittal E14.5 mouse section, showing enrichment in the ventricular and sub-ventricular zones (arrowheads) relative to the cortical plate (CP). Images are from www.genepaint.org (Visel et al. 2004). (B) Representative PCR genotyping result from Emx1-Cre (control) and Emx1-Cre;Eif4a3lox/+ mice. Note a single band (432 bp) in control and two bands (432 bp and 490 bp) in Emx1-Cre;Eif4a3lox/+. (C) Representative western blot of Pax6. (D) Quantification of Pax6 expression from E10.5 cortical lysates. Error bars, S.D, n = 3 biological replicates each. (TIF) [file pgen.1006282.s001.tif]

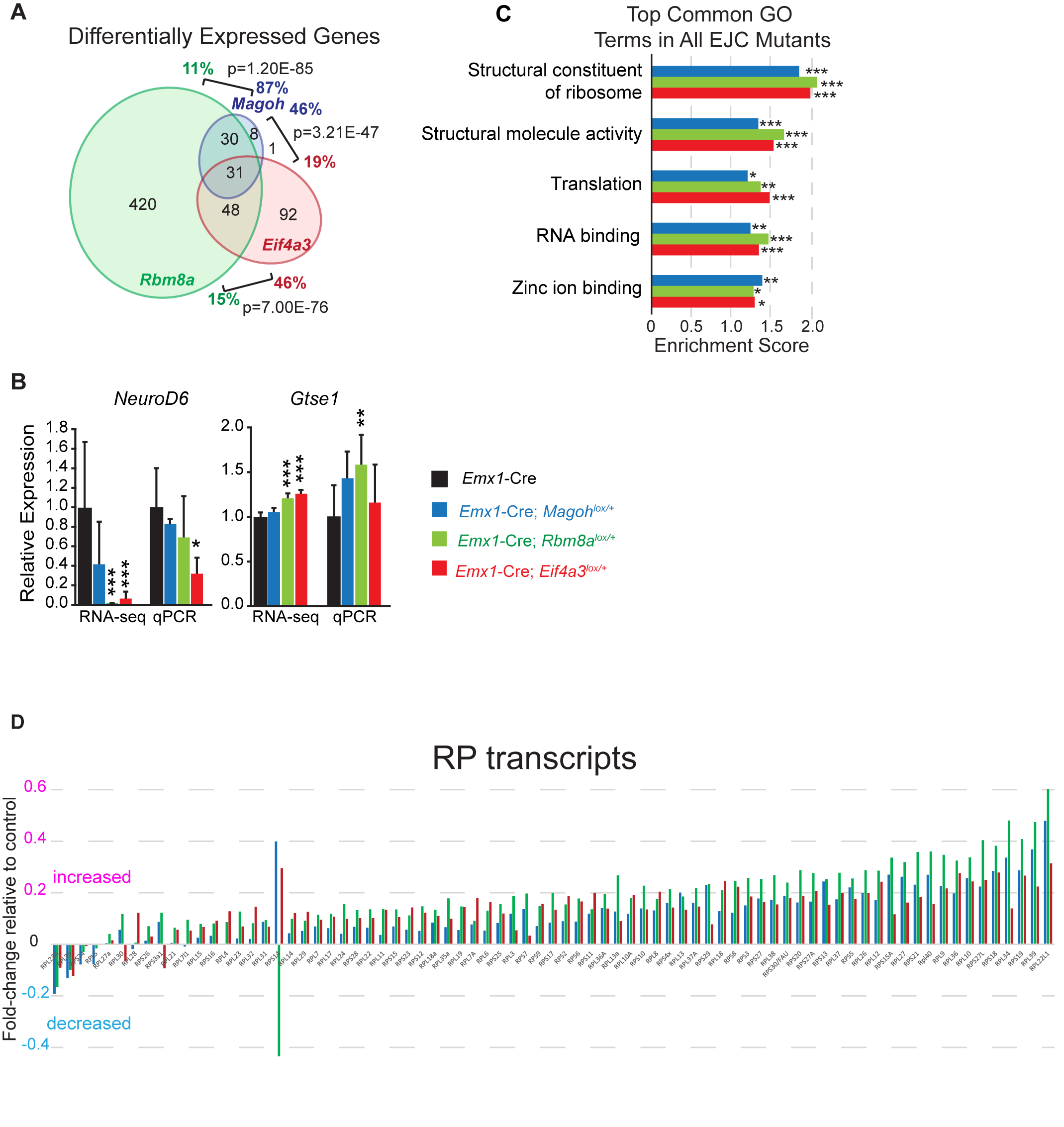

Supplement: S2 Fig — (A) Venn Diagrams showing the overlap of significant transcript changes (q<0.05) among the 3 EJC mutants. For pairwise comparisons between datasets, the percent of overlapping transcript changes within each mutant is shown, along with associated p values. (B) qPCR validation of NeuroD6 and Gtse1 mRNA expression in indicated E11.5 mutant cortices. For RNA-seq and qPCR, each control was normalized to 1.0 and compared to mutants. (C) Bar graph of top common enriched GO terms identified with GSEA analysis among all 3 EJC mutants, showing corresponding fold enrichment and P values. (D) Plot of all ribosomal protein transcripts for EJC mutant RNA seq. Student’s t-test (B). Error bars, S.D, *, p<0.05, **, p<0.01, ***, p<0.001. (TIF) [file pgen.1006282.s002.tif]

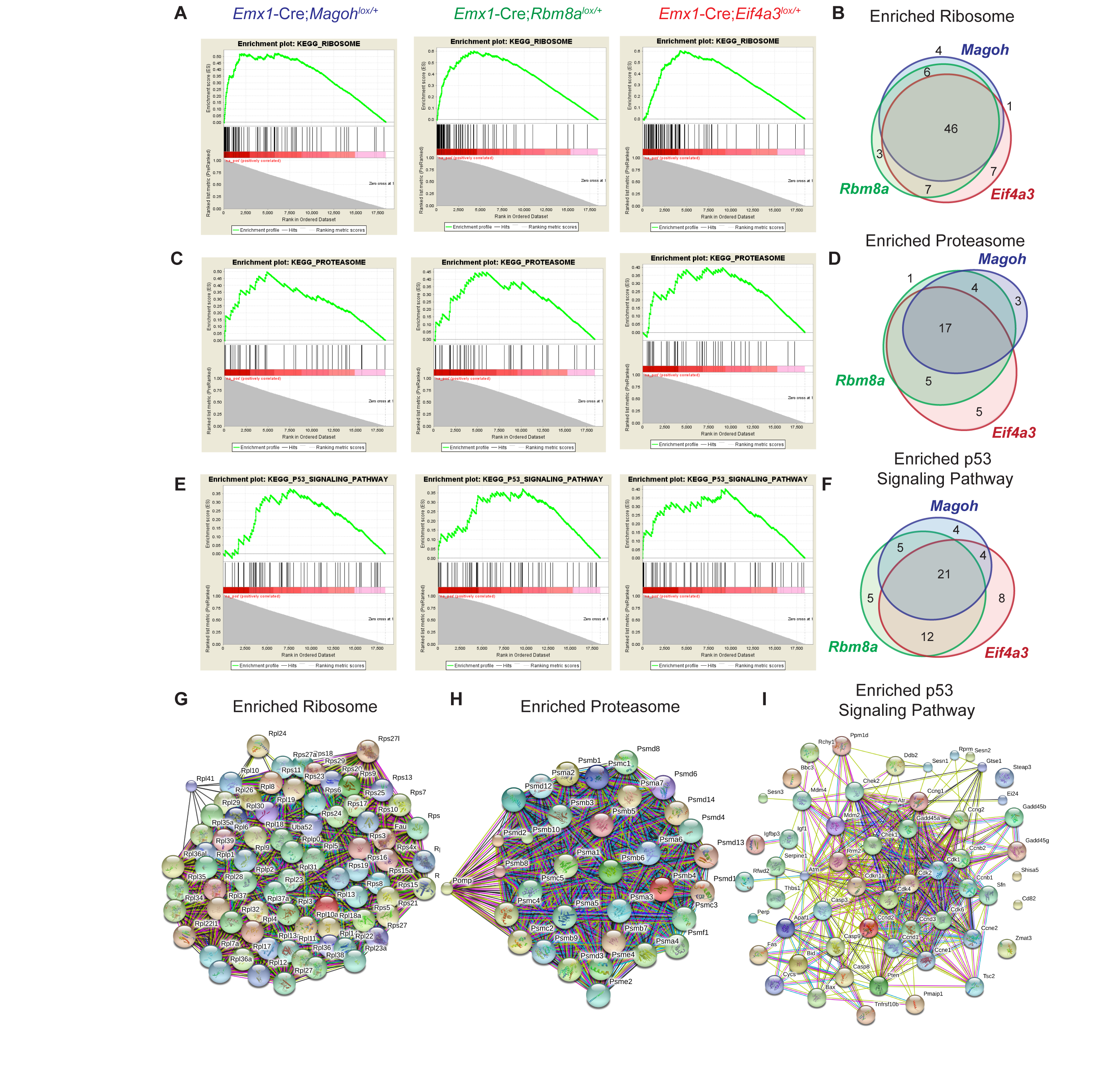

Supplement: S3 Fig — (A, C, E) Enrichment plots from GSEA KEGG analysis for Ribosome (A), Proteasome (C) and p53 signaling (E) terms. (B, D, F) Venn diagrams of overlapping enriched genes between EJC mutants for the Ribosome (B), Proteasome (D), and p53 signaling (F) terms. (G, H, I) STRING analysis of the genes enriched in the Ribosome (G), Proteasome (H), and p53 signaling (I) pathways. (TIF) [file pgen.1006282.s003.tif]

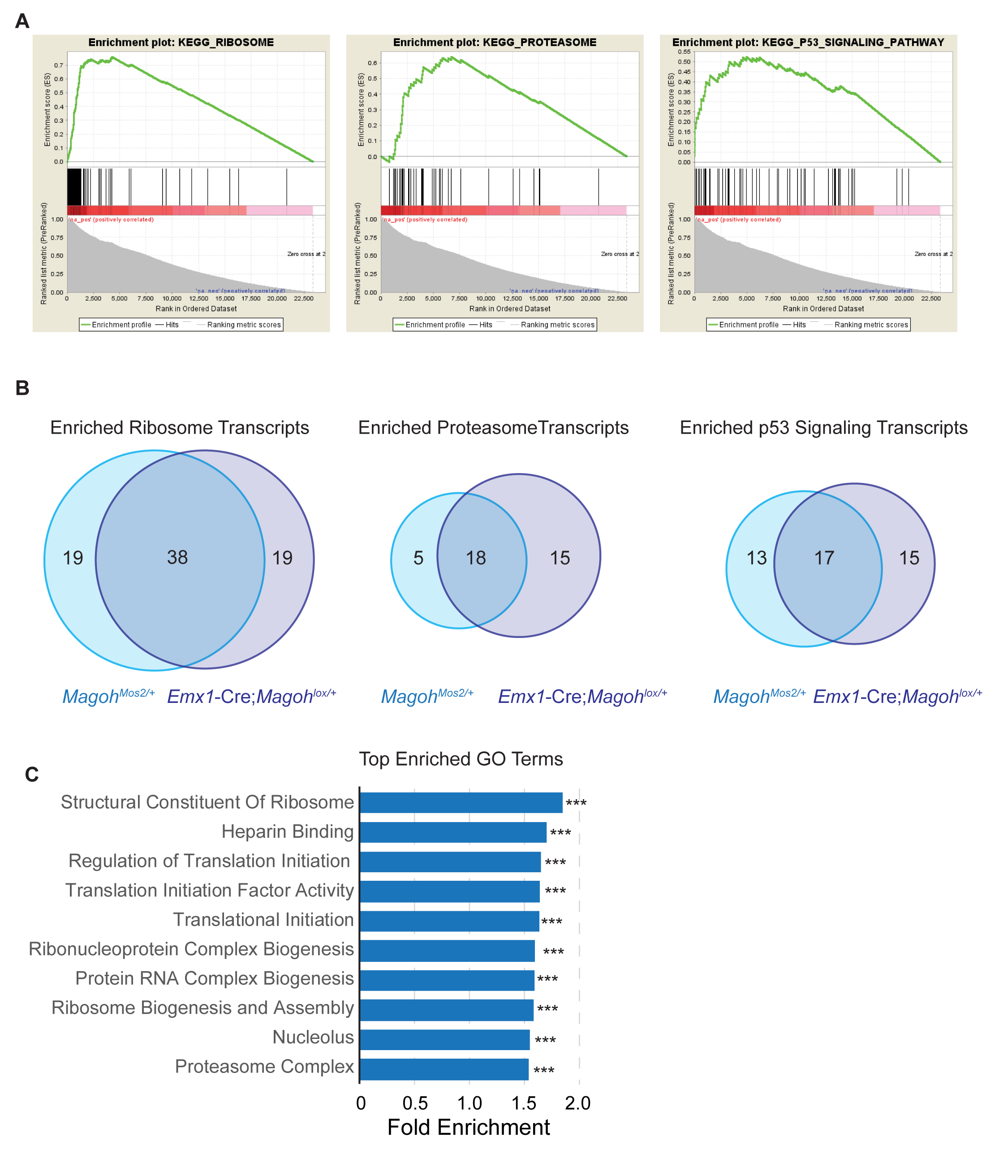

Supplement: S4 Fig — (A) Enrichment plots from GSEA KEGG analysis for Ribosome, Proteasome, and p53 signaling terms. (B) Venn diagrams of overlapping enriched genes between Emx1-Cre;Magohlox/+ and MagohMos2/+ mutants for the Ribosome, Proteasome, and p53 signaling terms. (C) Top enriched GO terms from GSEA analysis of MagohMos2/+ transcriptome. ***, p<0.001. (TIF) [file pgen.1006282.s004.tif]

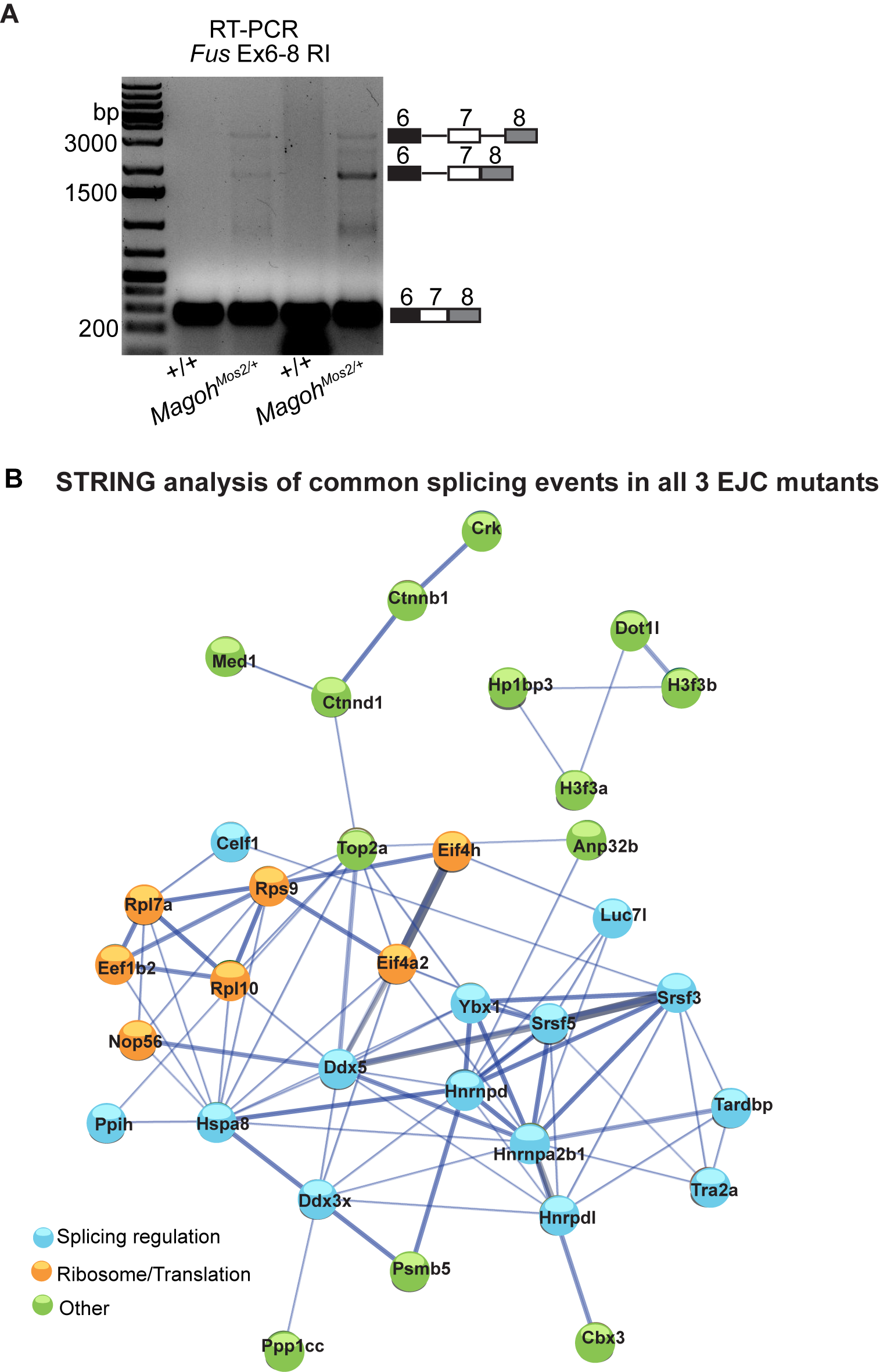

Supplement: S5 Fig — (A) Gel image showing detection of increased Fus Ex6-8 RI events in E11.5 MagohMos2/+ mutants compared to 2 litter mate controls. (B) STRING analysis including genes predicted to show identical, significant splicing changes in all 3 EJC mutants. Common genes that are not connected with any other genes by STRING analysis were not included. Stronger associations are represented by thicker lines. Note two networks of splicing regulation (cyan) and Ribosome/Translation (orange). (TIF) [file pgen.1006282.s005.tif]

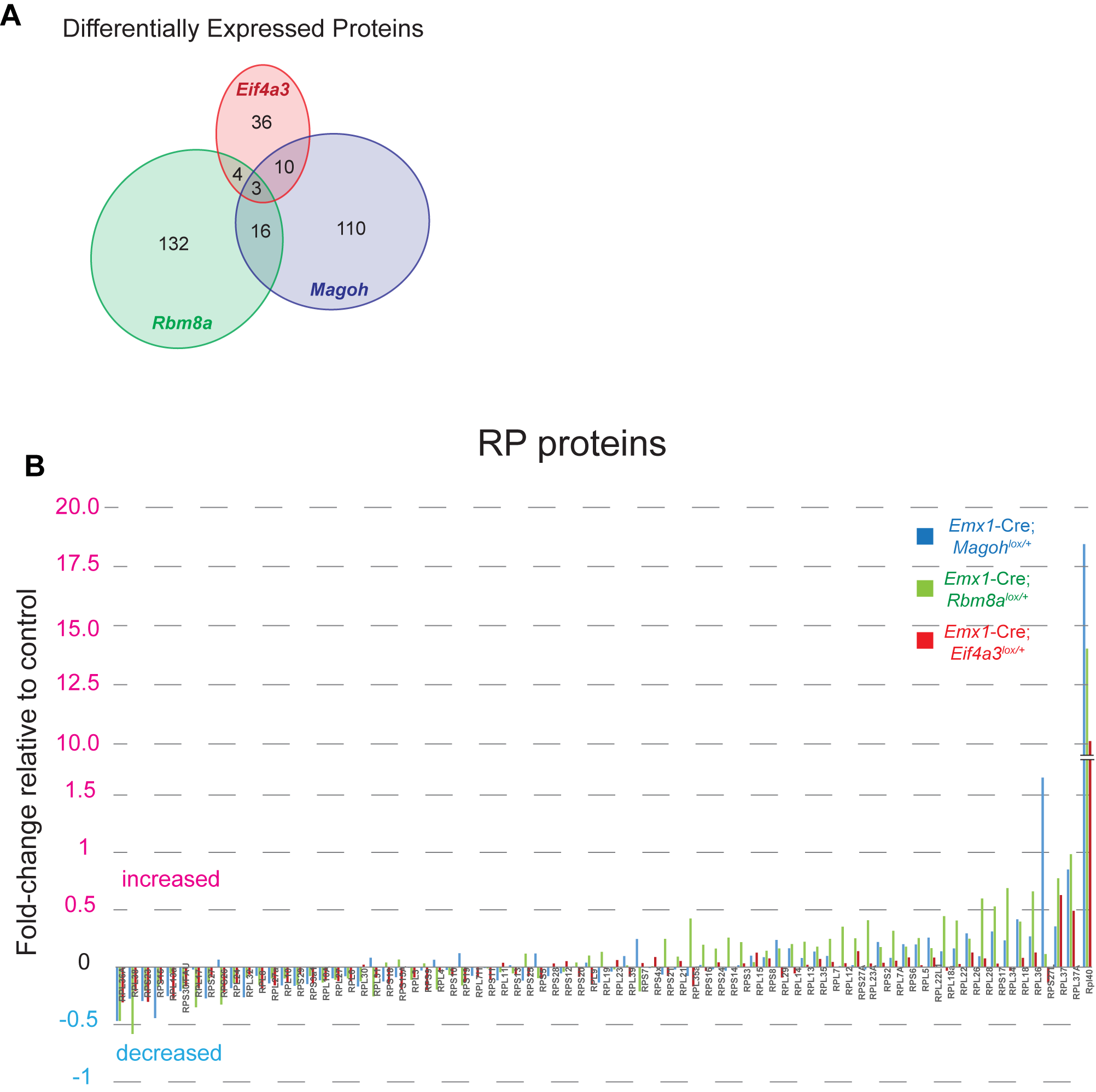

Supplement: S6 Fig — (A) Venn diagrams of overlapping enriched proteins altered between EJC mutants (p<0.05). (B) Bar graph depicting all ribosomal protein changes relative to control showing similar trends in all 3 EJC mutants (control levels are set to 0). (TIF) [file pgen.1006282.s006.tif]

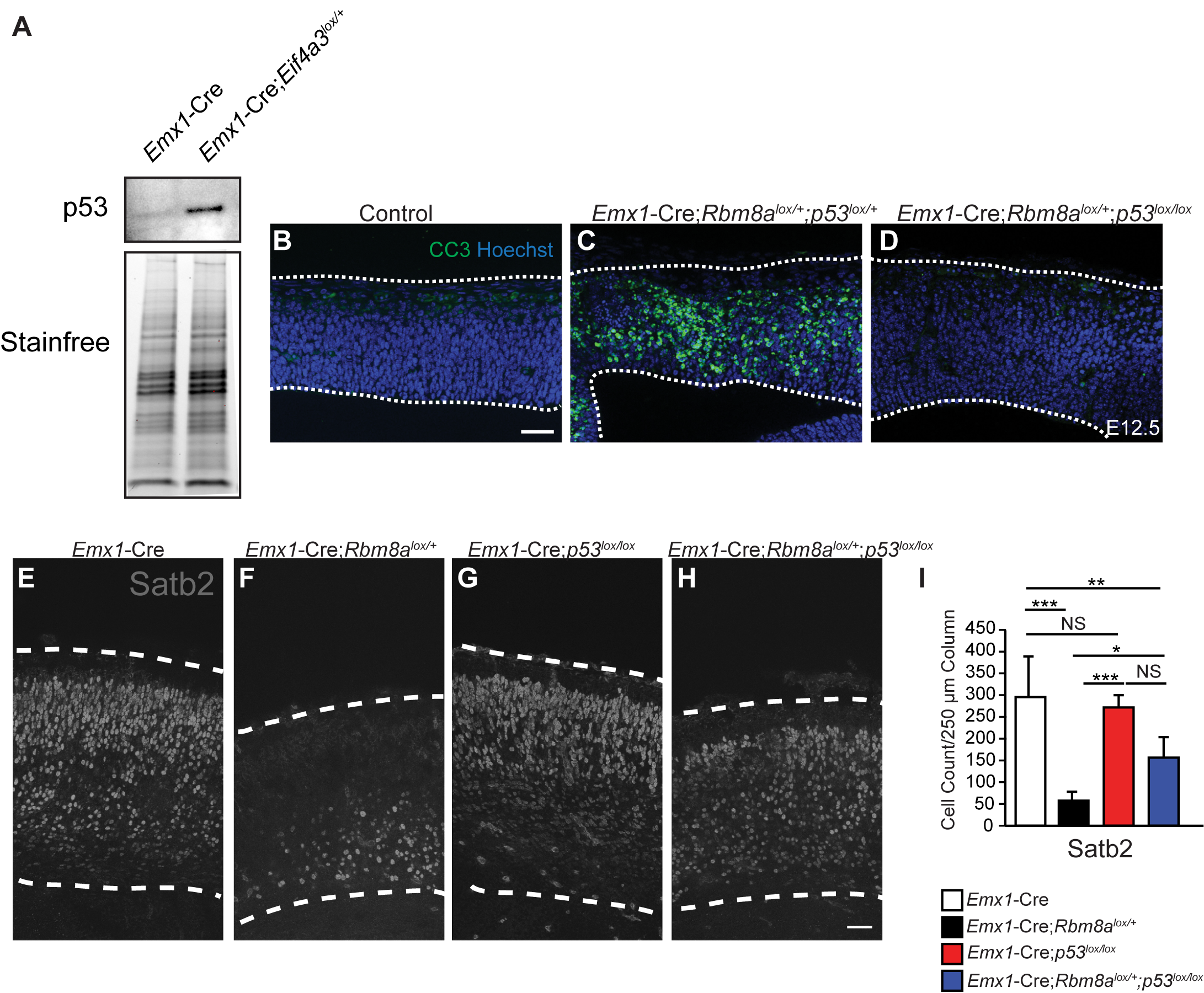

Supplement: S7 Fig — (A) Representative western blot for p53 in Emx1-Cre and Emx1-Cre;Rbm8alox/+ E11.5 dorsal cortex. (B-D) Immunostaining for CC3 in control (B), Emx1-Cre;Rbm8alox/+;p53lox/+ (C) and Emx1-Cre;Rbm8alox/+;p53lox/lox (D). (E-H) Representative images of Satb2 immunostaining in E18.5 cortex from Emx1-Cre, Emx1-Cre;Rbm8alox/+, Emx1-Cre;p53lox/lox and Emx1-Cre;Rbm8alox/+;p53lox/lox. (I) Quantification of Satb2+ cells from E-H. ANOVA with Tukey posthoc. Error bars, S.D. *,p<0.05, **,p<0.01, ***,p<0.001, Scale bar, 50 μm. (TIF) [file pgen.1006282.s007.tif]
